# The discovery of monoamine oxidase inhibitors: virtual screening and in vitro inhibition potencies

**DOI:** 10.1007/s10822-026-00764-y

**Published:** 2026-01-31

**Authors:** Maryké Shaw, Anél Petzer, Chantalle Crous, Theunis T. Cloete, Jacobus P. Petzer

**Affiliations:** 1 Centre of Excellence for Pharmaceutical Sciences, North-West University, Private Bag X6001, Potchefstroom, 2520 South Africa; 2 Pharmaceutical Chemistry, School of Pharmacy, North-West University, Private Bag X6001, Potchefstroom, 2520 South Africa

**Keywords:** Virtual screening, Monoamine oxidase, MAO, Docking, Guanabenz, Proflavine

## Abstract

The monoamine oxidase (MAO) enzymes are mitochondrial flavoenzymes that catalyse the oxidative deamination of neurotransmitter amines such as serotonin, norepinephrine and dopamine. Inhibitors of the MAOs are well-known antidepressant and antiparkinsonian agents, and act by reducing MAO-mediated metabolism of neurotransmitters in the brain. The present study attempted to identify compounds that inhibit the MAOs by virtual screening of existing drugs listed in the DrugBank using the Discovery Studio life science software. To identify the combinations of docking and scoring functions that most accurately identify known MAO inhibitors, the enrichment factor (EF^10%^) and area under the receiver operating characteristic curve (ROC-AUC) were evaluated. As a third validation metric, ligands that have been complexed with the MAOs were redocked and the root mean square deviation (RMSD) from the co-crystallized orientation was measured. The LibDock/LigScore 2 combination yielded the best results for both MAO-A (EF^10%^: 5.20, ROC-AUC: 0.82) and MAO-B (EF^10%^: 7.47, ROC-AUC: 0.89). Among the top 100 hits, ten compounds were selected and evaluated as in vitro inhibitors of human MAO. Guanabenz (IC_50_ = 3.46 µM) and proflavine (IC_50_ = 0.223 µM) were found to be the most potent MAO-A inhibitors. These compounds also inhibited MAO-B with IC_50_ values of 8.49 and 34.3 µM, respectively. Kinetic analysis showed a competitive mode of MAO-A inhibition for guanabenz (K_i_ = 0.16 µM) and proflavine (K_i_ = 0.066 µM). These results show that the validated virtual screening protocol is a useful tool to aid in the discovery of MAO inhibitors.

**Graphical abstract:**

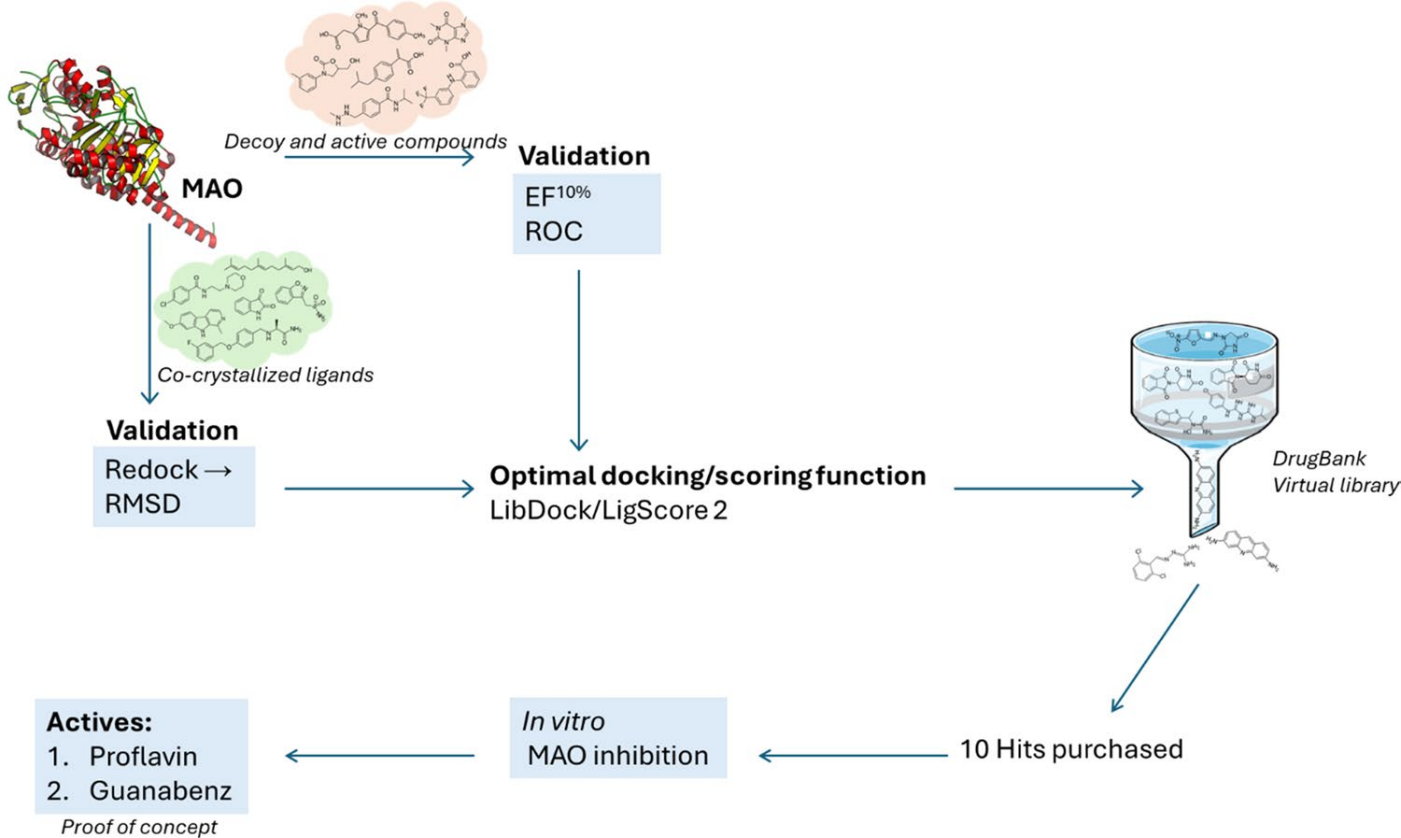

**Supplementary Information:**

The online version contains supplementary material available at 10.1007/s10822-026-00764-y.

## Introduction

The monoamine oxidase (MAO) enzymes are mitochondrial outer membrane flavoproteins that exist as two distinct isoforms, MAO-A and MAO-B [1, 2]. The primary physiological function of MAO is to catalyse the oxidative deamination of neurotransmitter amines in the brain and peripheral tissues, thereby terminating their actions [3]. Despite their structural similarities, the MAOs have different substrate and inhibitor specificities. MAO-A displays specificity for serotonin as substrate while MAO-B specifically metabolizes the arylalkylamines, benzylamine and phenethylamine [4]. Noradrenaline, adrenaline and dopamine are substrates for both isoforms.

**Fig. 1:**
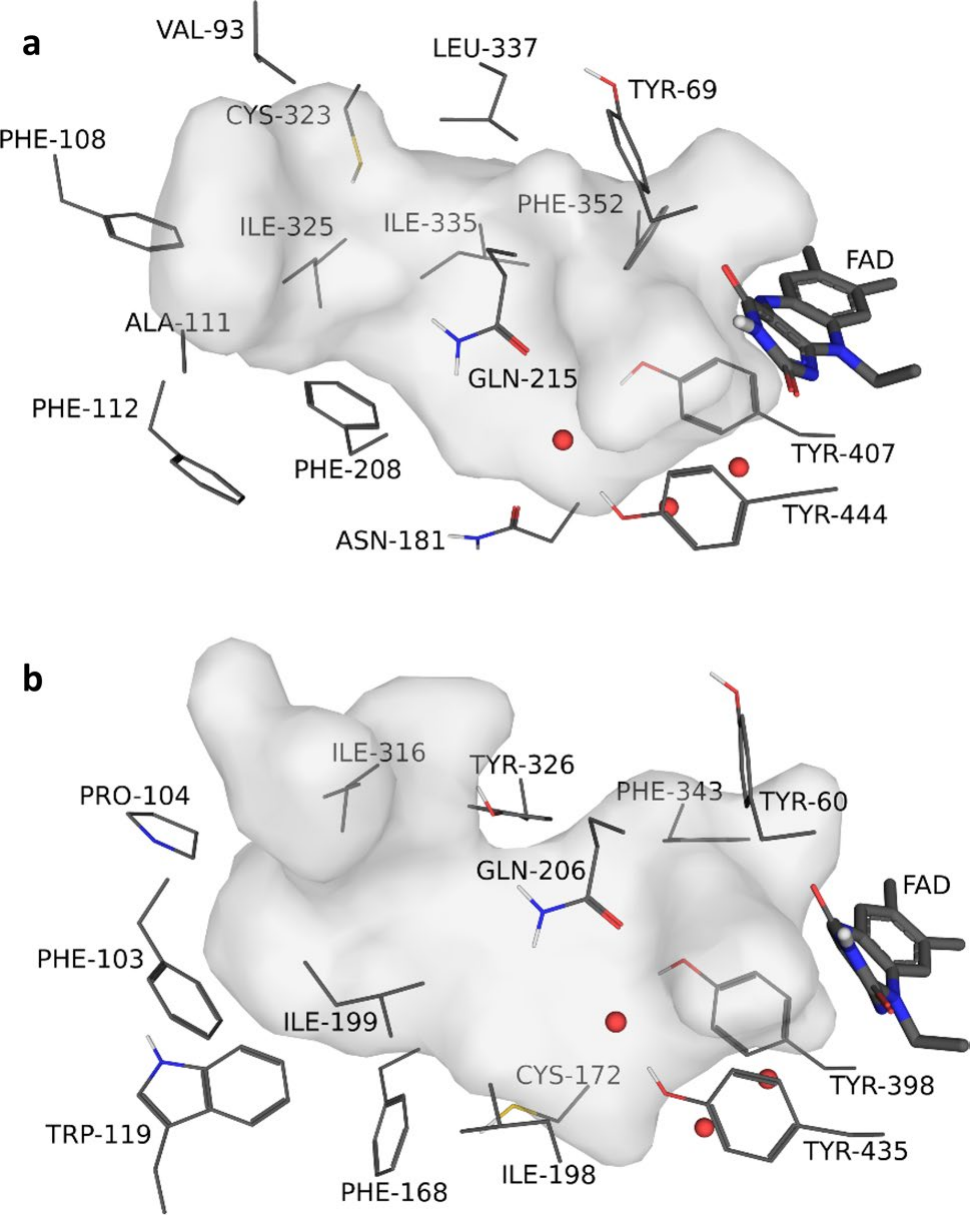
The active sites of MAO-A (panel**a**) and MAO-B (panel** b**) with the cavities shown as grey surfaces. For clarity, the MAO-A and MAO-B structures are presented with the same viewing angles, the FAD cofactors are shown as sticks, the residues lining the active sites are shown as lines and the water molecules are indicated by red spheres. The tyrosine residues forming the aromatic cage are: Tyr407/Tyr444 in MAO-A; Tyr398/Tyr435 in MAO-B

The availability of the three-dimensional structures of human MAO-A and MAO-B has greatly enhanced our understanding of the structural features that small molecules should possess for potent inhibition (Fig. 1) [5–7]. The human MAO isoforms are similar in their three-dimensional structures, and in their active sites, key amino acid residues are conserved. In this respect, of the 16 residues that line the MAO-A active site, only six differ between MAO-A and MAO-B [7]. The active site of MAO-A consists of a single cavity with a volume of ~ 550 Å^3^ while the MAO-B active site is comprised of a substrate cavity and an entrance cavity with volumes of ~ 420 and ~ 290 Å^3^, respectively [5, 6]. The two cavities in MAO-B are separated by the side chains of Tyr326, Ile199, Leu171 and Phe168. The side chain of Ile199 acts as a gate and can rotate from the active site to fuse the two cavities into a larger single cavity. For both isoforms, an aromatic cage environment, which serves as substrate recognition site, is formed by two tyrosine residues (Tyr407/Tyr444 in MAO-A; Tyr398/Tyr435 in MAO-B) and the flavin of the flavin adenine dinucleotide (FAD). The difference in inhibitor specificities between MAO-A and MAO-B has been attributed to two amino acid substitutions in the active sites of the MAOs: Phe208 and Ile335 in MAO-A are replaced by Ile199 and Tyr326 in MAO-B (e.g., PDB codes 2Z5X and 2BK3) [7, 8]. The binding of MAO-A-specific inhibitors such as harmine is prevented by steric overlap with Tyr326 in MAO-B. Conversely, the binding of MAO-B-specific inhibitors such as safinamide is hampered by an unfavourable interaction with Phe208 in MAO-A (e.g., PDB codes 2Z5X and 2V5Z) [9]. The residue in MAO-B that corresponds to Phe208 in MAO-A is Ile199. As mentioned, the side chain of Ile199 can rotate from the active site cavity allowing larger inhibitors to bind to MAO-B. Such inhibitors are known as cavity-spanning inhibitors and bind to both substrate and entrance cavities of MAO-B.

Based on their roles in the metabolism of neurotransmitters, the MAOs are of relevance to several neuropsychiatric and neurodegenerative disorders such as depression, Alzheimer’s disease and Parkinson’s disease [10–15]. The present study attempted to discover novel inhibitors of MAO-A and MAO-B by following a virtual screening approach [16]. Existing drugs listed in the DrugBank were docked into selected crystal structures of MAO-A and MAO-B using the Discovery Studio life science software. Three docking algorithms, CDocker, LibDock and LigandFit were used for the docking and the docked orientations were scored with 11 different scoring functions. The docking algorithm finds the most likely orientations that a ligand will adopt in the active site while the scoring function evaluates the interactions between the ligand and active site and predict the binding affinities of the different orientations [17]. However, the virtual screening procedure was firstly validated to determine the combinations of docking and scoring functions that most accurately identified active MAO inhibitors. For the validation, the enrichment factor (EF^10%^) and area under the receiver operating characteristic curve (ROC-AUC) were evaluated, while ligands present in X-ray crystal structures of the MAOs were redocked and the root mean square deviation (RMSD) from the co-crystallized orientation was measured [16, 18, 19]. Using the optimal combination, the compounds in the DrugBank were docked and scored, and the highest ranked solutions were retrieved. Among the 100 highest ranked drugs, ten were purchased and evaluated as in vitro inhibitors of human MAO-A and MAO-B. The approach of mining a database of existing and registered drugs for a new indication is known as drug repurposing or drug repositioning [20, 21]. The advantage of drug repurposing is that the safety and pharmacokinetic profiles of the drugs have been evaluated in human trials, which might simplify the process of registering the drug for a new indication.

While many experimental MAO inhibitors have been described, few studies on the investigation of current medications as potential MAO inhibitors have been reported. Following a computational approach to discover MAO inhibitors among existing drugs has been shown to be effective, and this approach was thus followed for the present study [22, 23]. Considering the availability of high-quality crystal structures for the MAO enzymes, a structure-based rather than ligand-based approach was followed. The hypothesis was that among the drugs listed in the DrugBank, some will exhibit MAO inhibition. Such drugs might be repurposed as MAO inhibitors or alternatively the discovery of their MAO inhibition properties might extend knowledge regarding their pharmacological and safety profiles.

## Experimental section

### Crystal structures of monoamine oxidase

For the computational studies, X-ray crystal structures of MAO-A and MAO-B were obtained from the RCSB Protein Data Bank (PDB). The structures were selected based on the presence of a non-covalently bound ligand in the active site (Table 1). For MAO-B, several structures have been reported and are available in the PDB, however, 21 structures complexed with non-covalently bound ligands were selected while one structure of MAO-A with a reversible inhibitor bound to the active site was retrieved. As detailed below, the co-crystallised ligands present in the MAO structures were redocked into a selected structure (e.g., 2Z5X for MAO-A; 2V5Z for MAO-B) and the RMSD from the co-crystallized orientation was measured [7, 9]. These selected protein structures (e.g., 2Z5X for MAO-A; 2V5Z for MAO-B) have been determined at high resolutions and were also used to validate the virtual screening procedure by evaluating EF^10%^ and ROC-AUC.

**Table 1:** The PDB codes of the MAO-A and MAO-B structures complexed with non-covalently bound ligands

PDB code of MAO-B structure	Co-crystallized ligand	Resolution (Å)
2V5Z [9]	Safinamide	1.6
1OJ9 [24]	1,4-Diphenyl-2-butene	2.3
1OJA [24]	Isatin	1.7
1OJD [24]	Lauryldimethylamine-N-oxide (LDAO)	3.1
2BK3 [8]	Farnesol	1.8
2V60 [9]	7-(3-Chlorobenzyloxy)-4-carboxaldehyde-coumarin	2
2V61 [9]	7-(3-Chlorobenzyloxy)-4-(methylamino)methyl-coumarin	1.7
3PO7 [25]	Zonisamide	1.8
4A79 [22]	Pioglitazone	1.89
4A7A [22]	Rosiglitazone	1.7
4CRT [26]	Multi-target inhibitor ASS234	1.8
5MRL [27]	N-(Furan-2-ylmethyl)-N-methylprop-2-yn-1-amine (F2MPA)	2.42
6FVZ [28]	Dimethylphenyl-chromone-carboxamide	1.8
6FW0 [28]	Chlorophenyl-chromone-carboxamide	1.6
6FWC [28]	Fluorophenyl-chromone-carboxamide	1.7
6RKB [29]	Styrylpiperidine analogue 1	2.3
6RKP [29]	Styrylpiperidine analogue 84	1.7
6RLE [29]	Styrylpiperidine analogue 97	2.3
6YT2 [30]	Diphenylene iodonium (DPI)	1.8
7B0V [31]	(*E*)-3-Phenyl-1-(3-(trifluoromethyl)phenyl)prop-2-en-1-one	2.3
7B0Z [31]	(*E*)-3-Phenyl-1-(4-(trifluoromethyl)phenyl)prop-2-en-1-one	2.1
**PDB Code of MAO-A structure**	**Co-crystallized ligand**	
2Z5X [7]	Harmine	2.20

These structures were selected for determining of RMSD values following redocking of the co-crystallized ligands into the 2Z5X and 2V5Z structures, respectively

### Protein structure preparation and docking

The life sciences software Discovery Studio V3.1 (Accelrys Inc., San Diego, CA, USA) was used for all computational studies. Unless otherwise specified, all parameters within Discovery Studio were set to their default values. The protein structures (e.g., 2Z5X for MAO-A; 2V5Z for MAO-B) that were selected for the molecular docking were firstly prepared by calculating the pKa values and protonation states of the ionisable amino acids after which hydrogen atoms were added at pH 7.4. If required, the valences of the FAD co-factors (oxidised state) and co-crystallised ligands were corrected, and the protein models were automatically typed with the Momany and Rone CHARMm forcefield. A fixed atom constraint was applied to the backbone and the structures were energy minimised using the Smart Minimizer algorithm with the maximum number of steps set to 50,000, employing the implicit generalized Born solvation model with molecular volume. The co-crystallised ligands (e.g., harmine in MAO-A and safinamide in MAO-B), waters and the backbone constraints were removed from the models and the binding sites were identified from an analysis of the enzyme cavities. In each model, three active site waters are considered to be conserved and were retained. These waters were HOH 710, 718 and 739 in the MAO-A active site, and HOH 1155, 1170 and 1351 in the A-chain of the MAO-B active site. Docking was carried out with the CDocker, LibDock and LigandFit algorithms. For CDocker, ten random ligand conformations were generated, the heating target temperature was set to 700 K and full potential mode was employed. For LigandFit the default values were applied, while the Conformation Method for LibDock was set to BEST [16, 32]. In addition to being scored with the native scoring functions of each docking algorithm (e.g., CDOCKER Energy, LibDock Score and Dock Score, respectively), the solutions were also rescored with the following ten functions: LigScore 1 & 2, PLP 1 & 2, Jain, PMF, PMF 4 and Ludi 1, 2 & 3. All illustrations were prepared with the PyMOL molecular graphics system (Schrödinger, Inc. New York, NY, USA) [33].

### Root mean square deviation

The 21 co-crystallised ligands (Table 1) were prepared for the docking by firstly correcting the valences where required, followed by the addition of hydrogen atoms. The geometries of the structures were briefly optimised using a Dreiding-like forcefield (5000 iterations) and the structures were submitted to the Prepare Ligands protocol. Atom potential types and partial charges were subsequently assigned to the structures with the Momany and Rone CHARMm forcefield and the structures were docked into the selected protein structures (e.g., 2Z5X for MAO-A; 2V5Z for MAO-B) as described above. The RMSD of the redocked orientation from the orientation of the ligand in the co-crystallised structure was measured and the success rate was calculated. Typically, orientations within an RMSD of 2 Å are considered successful, and the success rate was thus defined as the percentage of ligands that were redocked with an RMSD < 2 Å [34]. However, some docking studies have used an RMSD of ≤ 1.5 Å as an indication of successful docking [19] (Table 2).

**Table 2:** RMSD as validation metric for redocking of co-crystallised ligands into the active site of MAO-B with *LibDock* and scoring the resulting poses with selected scoring functions. ^a^

	Total^c^	Success rate (%)^d^
Best pose^b^	19	90
*Scoring function*		
Native^e^	8	38
LigScore 1	6	29
LigScore 2	8	38
PLP 1	5	24
PLP 2	3	14
Jain	1	5
PMF	7	33
PMF 4	8	38
Ludi 1	6	29
Ludi 2	2	10
Ludi 3	0	0

^a^The complete table is given in the supplementary (Table S3)

^b^Refers to the best pose obtained for each ligand among all the poses that were generated

^c^Number of ligands with an RMSD value < 2 Å (from a total of 21 ligands)

^d^Percentage of ligands with and RMSD value < 2 Å

^e^Native scoring function for LibDock – LibDock Score

### Enrichment factor

Twenty-three known inhibitors of MAO-A and 24 known MAO-B inhibitors were obtained from literature (Table S1, supplementary) and were submitted to the Directory of Useful Decoys: Enhanced (DUD-E, https://dude.docking.org) [35]. For each ligand, 50 to 100 decoy compounds were generated, and in total 2543 decoy compounds were retrieved for both MAO-A and MAO-B and imported into Discovery Studio. DUD-E decoys are matched to the ligands by the following properties: molecular weight, logP, number of rotatable bonds, hydrogen bond donors and acceptors, and net charge. For comparison with the DUD-E, the Schrödinger drug-like decoy set of drug-like compounds (average molecular weight of 400), which contains 999 decoy compounds, was also used in this study [19]. The known MAO inhibitors (Table S1), which are considered the active inhibitors, were imported into both decoy databases. The resulting databases containing active and decoy compounds were prepared for docking as described above for the determination of RMSD values. These databases were docked with LibDock and LigandFit into the selected protein structures (e.g., 2Z5X for MAO-A; 2V5Z for MAO-B) and scored with the native scoring functions as well as the ten additional scoring functions as described above. For each combination of docking and scoring function, the EF^10%^ was calculated in Microsoft Excel according to Eq. 1. The EF^10%^ represents the proportion of the active compounds that is found in the 10% highest ranked compounds of the entire database [36–39].

1 $$\:{EF}^{10\%}=\left(\frac{{Hits}_{sel}}{{Hits}_{tot}}\right)\times\:\left(\frac{{NC}_{tot}}{{NC}_{sel}}\right)$$

Hits_sel_ is the number of active compounds present in the 10% highest ranked compounds; Hits_tot_ is the total number of active compounds present in the entire database; NC_tot_ is the sum of the active and decoy compounds present in the entire database; NC_sel_ is the is the sum of the active and decoy compounds present in the 10% highest ranked compounds. The maximum possible EF^10%^ is equal to 10, the inverse of the percentage (10%) of the highest ranked compounds considered for the database.

### ROC-AUC

The ROC curve is a graph of the true positive fractions (TPF or sensitivity, y-axis) versus false positive fractions (FPF or 1 – specificity, x-axis) for all compounds in the database [38, 40]. For a scoring function with the ability to discriminate between active and decoy compounds, the curve passes through the upper left corner of the graph and the AUC would be closer to 1. The DUD-E and Schrödinger drug-like decoy datasets containing the known MAO-A and MAO-B inhibitors that were prepared above, were used to construct ROC curves using SPSS Statistics for Windows V27 (IBM, Armonk, NY, USA). For each combination of docking and scoring function, the ROC-AUC was calculated and interpreted as follows: 0.9 ≤ AUC ≤ 1.0 is excellent, 0.8 ≤ AUC *<* 0.9 is good, 0.7 ≤ AUC *<* 0.8 is fair, 0.5 ≤ AUC *<* 0.7 is poor and AUC *<* 0.5 is a failure [41].

### Monoamine oxidase inhibition

MAO inhibitory potencies of the test inhibitors were determined according to the procedure reported in literature [32, 42]. Kynuramine served as non-specific MAO substrate and commercially available recombinant human MAO-A and MAO-B served as enzyme sources (Merck, Darmstadt, Germany). MAO is supplied as a membrane suspension of recombinant MAO expressed in baculovirus infected insect cells. Kynuramine is oxidised by MAO-A and MAO-B to ultimately yield 4-hydroxyquinoline which was measured by fluorescence spectrophotometry (λ_ex_ = 310 nm; λ_em_ = 400 nm) and the resulting concentration data were used to calculate the MAO catalytic rates. The catalytic rates were thus measured in the presence and absence of a range of inhibitor concentrations (0.003–100 µM) and sigmoidal graphs of catalytic rate versus the logarithm of inhibitor concentration (log[I]) were constructed by fitting the data to the one-site competition model with the Prism 5 software package (GraphPad Software Inc., Boston, MA, USA). The IC_50_ values were determined from these graphs and were reported as the mean ± standard deviation (SD) of triplicate experiments. To verify that the test inhibitors, at 10 and 100 µM, do not interfere with the fluorescence under the experimental conditions, their effect on the fluorescence of 4-hydroxyquinoline (0.75 µM) was investigated.

This experimental procedure was also used to construct Lineweaver-Burk graphs for the inhibition of MAO-A by proflavine and guanabenz. For these experiments the concentration ranges of the inhibitors were 0.0558–0.279 µM for proflavin and 0.865–4.325 µM for guanabenz while the substrate concentrations ranged from 15 to 250 µM. The enzyme-inhibitor dissociation constant (K_i_) was estimated from a replot of the slopes of the Lineweaver-Burk plots versus inhibitor concentration where the x-axis intercept equals –K_i_. Linear regression analysis was carried out with the Prism 5 software.

### Virtual screening of the DrugBank

A virtual library of the compounds listed in the DrugBank was obtained from the DrugBank and prepared in Discovery Studio for the docking by firstly adding hydrogen atoms, optimising the geometries of the structures using a Dreiding-like forcefield (5000 iterations) and submitting the structures to the Prepare Ligands protocol. The most promising docking and scoring combination was found to be LibDock and LigScore 2 as described below. The LibDock/LigScore 2 combination was selected based on the RMSD, EF^10%^ and ROC-AUC metrics. Atom potential types and partial charges were subsequently assigned to the structures with the Momany and Rone CHARMm forcefield and the structures were docked into the selected MAO protein structures using LibDock as described above. Following the docking, the solutions were rescored with the LigScore 2 function. The 100 highest ranked solutions for both MAO-A and MAO-B were retrieved.

## Results and discussion

### Validation—root mean square deviation

The RMSD was used as a validation metric to determine if a docking algorithm could correctly predict the position and orientation of a ligand in the MAO-A and MAO-B active sites as well as to determine if a specific scoring function had the ability to identify correct poses as indicated by an RMSD < 2 Å. Based on literature, an RMSD within 2 Å indicates that the orientation and position of a ligand have been correctly reproduced by the docking algorithm [34]. From these data the combination of docking algorithm and scoring function with the highest accuracy and best ability at identifying correct orientations, was determined. The 21 co-crystallised ligands (Table 1) were docked with CDocker, LibDock and LigandFit into the selected structures of MAO-B and one into MAO-A, after which the RMSD from the position of the co-crystallised ligand was determined. CDocker and LigandFit produced 10 poses for each ligand, while LibDock produced 12–100 poses. The poses were rescored with the native scoring function of each docking algorithm as well as the ten additional scoring functions.

The *best pose* column (column 2) in each table shows the lowest RMSD value that was obtained for each ligand among all the poses that were generated for that ligand with the pose number given in brackets (Table 2, S2, S3, S4). For MAO-B, CDocker (Table S2) and LibDock (Table 2 and S3) had a success rate of 62 and 90%, respectively, while LigandFit (Table S4) displayed a success rate of 43%, failing to redock four ligands into the MAO-B active site. LibDock gave the lowest average RMSD of 1.4 Å. These results showed that LibDock performed best at correctly redocking the co-crystallised ligands within the number of poses generated. The *scoring functions* column (columns 3–13) in each table shows the RMSD value of the pose that was ranked highest by a particular scoring function for each ligand. Here LigScore 2, PLP 1, Jain and PMF 4 performed best when docking was carried out with CDocker, with a success rate of 48% (Table S2). When docking was carried out with LibDock, the native scoring function (LibDock Score), LigScore 2 and PMF 4 performed best at ranking a successful pose first, with a success rate of 38% (Table 2 and S3). For LigandFit, the PMF scoring function performed best with a success rate of 38%, taking in account that this docking algorithm was unable to dock four ligands into the MAO-B active site (Table S4). The results thus showed that CDocker combined with several scoring functions (e.g., LigScore 2, PLP 1, Jain and PMF 4) yielded the highest success rates for correctly redocking the co-crystallised ligands and ranking successful poses (e.g., those with RMSD < 2 Å) first. LibDock combined with the native scoring function, LigScore 2 and PMF 4 also gave accurate results with slightly lower success rates than the best performing scoring functions combined with CDocker.

A similar analysis could not be carried out for MAO-A, since only one structure with a reversible bound co-crystallised ligand has been reported (Table 1). However, the ligand was redocked into the MAO-A active site with CDocker, LibDock and LigandFit, and the resulting poses were scored with the native scoring functions of each docking algorithm as well as the ten additional scoring functions. The results are presented in Table 3 and show that the following combinations successfully redocked the ligand and ranked successful poses (e.g., those with RMSD < 2 Å) first: CDocker/LigScore 2, CDocker/Ludi 1 & 3, LibDock/LigScore 2 and LigandFit combined with all scoring functions. It is noteworthy that the LibDock/LigScore 2 combination gave accurate results for both MAO-A and MAO-B.

**Table 3:** RMSD as validation metric for redocking of the co-crystallised ligand into the active site of MAO-A (PDB code: 2Z5X) with CDocker, LibDock and LigandFit, and scoring the resulting poses with selected scoring functions

Docking algorithm	CDocker	LibDock	LigandFit
Best pose (rank)	0.5 (8)	0.4 (15)	0.5 (6)
Native − 1st^a^	2.9	3.2	0.5
LigScore 1–1st (rank)	2.9 (2)	6.0 (3)	0.6 (4)
LigScore 2–1st (rank)	0.5 (9)	0.9 (10)	0.7 (10)
PLP 1–1st (rank)	2.9 (2)	2.6 (2)	0.6 (3)
PLP 2–1st (rank)	2.9 (2)	2.6 (2)	0.6 (4)
Jain − 1st (rank)	2.9 (3)	6.0 (3)	0.5 (6)
PMF − 1st (rank)	2.9 (2)	2.6 (2)	0.5 (6)
PMF 4–1st (rank)	2.9 (3)	2.6 (2)	0.6 (5)
Ludi 1–1st (rank)	0.5 (8)	3.0 (28)	0.6 (4)
Ludi 2–1st (rank)	2.9 (1)	6.0 (21)	0.6 (4)
Ludi 3–1st (rank)	0.6 (5)	6.0 (23)	0.6 (4)

^a^Native scoring function for CDOCKER – CDOCKER Energy; LibDock – LibDock Score; LigandFit – Dock Score

### Validation—enrichment factor and ROC

The EF^10%^ and ROC-AUC were used as validation metrics to determine which combinations of a docking algorithm and scoring function can most accurately identify active MAO inhibitors in a database that contains both active and inactive compounds (e.g., decoys). As detailed in the experimental section, 23 known inhibitors of MAO-A and 24 known MAO-B inhibitors were seeded into the DUD-E (*n* = 2543) and Schrödinger (*n* = 999) decoy datasets. The resulting databases were docked into the selected protein structures of MAO-A and MAO-B with LibDock and LigandFit and the poses were scored with native scoring function of each docking algorithm as well as the ten additional scoring functions. Using these data, the EF^10%^ and ROC-AUC were calculated. CDocker was not used as docking algorithm since it is computationally too expensive to use with large databases such as the decoy dataset used here and the DrugBank database that was used for virtual screening.

**Table 4:** EF^10%^ and ROC-AUC values as validation metrics for identifying 24 known MAO-B inhibitors within the DUD-E and Schrödinger decoy datasets. Docking was carried out with *LibDock* and the resulting poses were scored with selected scoring functions

	LibDock		LibDock	
	DUD-E^b^		Schrödinger^c^	
	EF^10%^	ROC-AUC	EF^10%^	ROC-AUC
Native^a^	3.32	0.844	1.66	0.475
LigScore 1	5.81	0.861	5.81	0.763
LigScore 2	7.47	0.888	7.47	0.865
PLP 1	3.74	0.872	2.08	0.591
PLP 2	3.74	0.852	1.25	0.542
Jain	0.00	0.332	0.42	0.362
PMF	4.57	0.861	1.66	0.535
PMF 4	2.49	0.813	2.08	0.566
Ludi 1	1.66	0.656	0.83	0.441
Ludi 2	0.42	0.547	0.42	0.435
Ludi 3	4.98	0.886	1.66	0.603

^a^Native scoring function: LibDock – LibDock Score

^b^Maximum EF^10%^ = 10.0 (2590 total compounds /24 active compounds)

^c^Maximum EF^10%^ = 10.0 (1046 total compounds /24 active compounds)

**Fig. 2:**
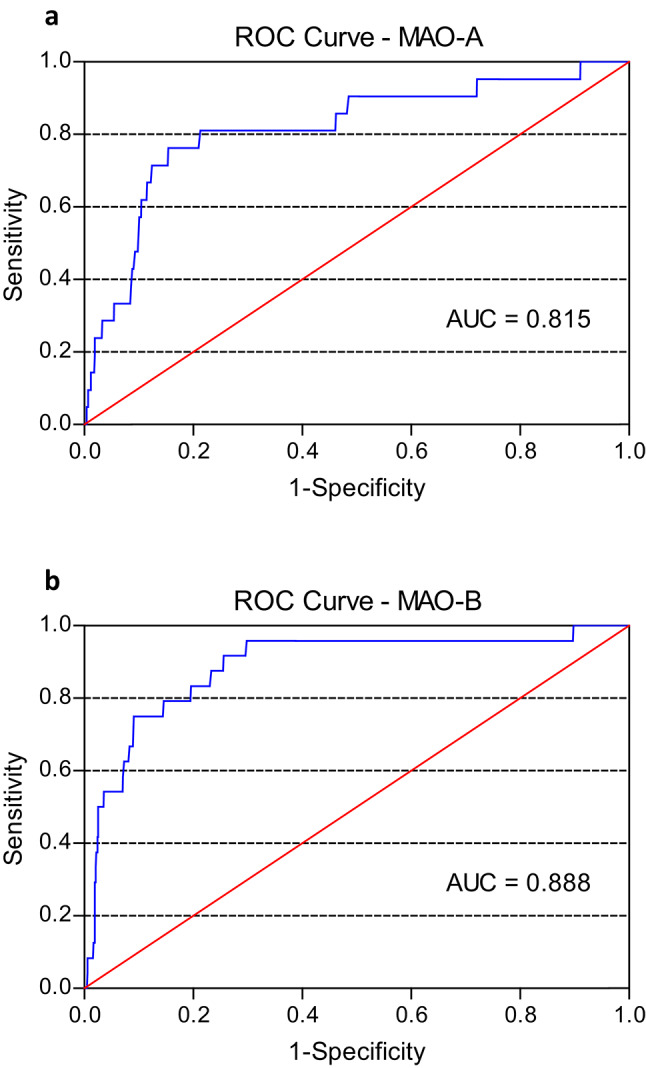
ROC curves for the identification of active MAO-A (panel **a**) and MAO-B (panel **b**) inhibitors in a database that contains both active and decoy compounds (e.g., DUD-E dataset). Docking was carried out with LibDock and the resulting poses were scored with LigScore 2

For MAO-B, the EF^10%^ values showed that LigScore 2 performed best with both the DUD-E (EF^10%^ = 7.47) and Schrödinger (EF^10%^ = 7.47) decoy datasets when using LibDock as docking algorithm (Table 4). With LigandFit as docking algorithm, LigScore 2 also performed best with both the DUD-E (EF^10%^ = 8.72) and Schrödinger (EF^10%^ = 8.72) decoy datasets (Table S5). With a maximum EF^10%^ of 10, these combinations thus performed well at discriminating active from decoy compounds. These results were supported by the ROC-AUC metric – for the LibDock/LigScore 2 combination, ROC-AUC values of 0.888 and 0.865 were recorded when using the DUD-E and Schrödinger decoy datasets, respectively (Fig. 2). For the LigandFit/LigScore 2 combination, ROC-AUC values of 0.897 and 0.887 were recorded when using the DUD-E and Schrödinger decoy datasets, respectively. These combinations thus possessed a very good accuracy and it may be concluded that they could be used to identify active MAO-B inhibitors in a database of compounds. It is worth mentioning that the combination of LigandFit and its native scoring function, Dock Score, was also found to be very accurate when using both the DUD-E (EF^10%^ = 8.72; ROC-AUC = 0.890) and Schrödinger (EF^10%^ = 8.72; ROC-AUC = 0.839) decoy datasets.

For MAO-A, similar results were obtained. The LibDock/LigScore 2 combination was most accurate for both the DUD-E (EF^10%^ = 5.20; ROC-AUC = 0.815) and Schrödinger (EF^10%^ = 7.36; ROC-AUC = 0.803) decoy datasets (Table 5). These ROC-AUC values indicated good accuracy and the ability of this combination to discriminate active from decoy compounds. For LigandFit, Ludi3 (EF^10%^ = 3.03; ROC-AUC = 0.804) was most accurate at discriminating between active and decoy compounds when using the DUD-E decoy dataset, while all combinations performed poorly when using the Schrödinger dataset (Table S6).

**Table 5:** EF^10%^ and ROC-AUC values as validation metrics for identifying 23 known MAO-A inhibitors within the DUD-E and Schrödinger decoy datasets. Docking was carried out with *LibDock* and the resulting poses were scored with selected scoring functions

	LibDock		LibDock	
	DUD-E^b^		Schrödinger^c^	
	EF^10%^	ROC-AUC	EF^10%^	ROC-AUC
Native^a^	1.30	0.512	0.43	0.313
LigScore 1	4.33	0.784	5.63	0.724
LigScore 2	5.20	0.815	7.36	0.803
PLP 1	1.30	0.496	0.43	0.348
PLP 2	2.17	0.51	2.17	0.384
Jain	0.00	0.262	0.43	0.277
PMF	0.87	0.534	2.17	0.424
PMF 4	0.00	0.521	1.30	0.484
Ludi 1	0.43	0.286	0.43	0.227
Ludi 2	0.43	0.191	0.43	0.217
Ludi 3	0.43	0.779	1.73	0.602

^a^Native scoring function: LibDock – LibDock Score

^b^Maximum EF^10%^ = 10.0 (2590 total compounds /23 active compounds)

^c^Maximum EF^10%^ = 10.0 (1046 total compounds /23 active compounds)

### Virtual screening of the Drugbank

Based on the RMSD, EF^10%^ and ROC-AUC metrics, the LibDock/LigScore 2 combination was deemed most accurate for both MAO-A and MAO-B, and was selected for virtual screening of the DrugBank. The compounds of the DrugBank were docked with LibDock into the selected structures of MAO-A and MAO-B and the resulting poses were scored with LigScore 2. Among the highest ranked 100 compounds for MAO-A and MAO-B, ten were selected and purchased (Table 6). Seven compounds were selected for MAO-A, two for MAO-B and one compound (proguanil) was predicted to be an inhibitor of both MAO-A and MAO-B. This selection was guided by the commercial availability of the highest ranked compounds. The coordinates of the ROC curve for the corresponding LigScore 2 values showed that the score of the lowest ranked drug that was selected as a potential MAO-A inhibitor (e.g., guanabenz, 4.92) corresponded to a TPF of 0.143 and a FPF of 0.017. The lowest ranked drug that was selected as a potential MAO-B inhibitor (e.g., chlorpropamide, 4.84) corresponded to a TPF of 0.542 and a FPF of 0.061. At these thresholds, 14.3 and 54.2% of the active inhibitors have been identified for MAO-A and MAO-B, respectively, while only 1.7 and 6.1% of inactive compounds were incorrectly identified as active inhibitors. Virtual screening is a common lead discovery approach and can deliver hit-rates that are significantly higher than high-throughput studies [19]. However, virtual screening is complementary to high-throughput screening and it should be recognised that active compounds may be overlooked by computational techniques where they would have been identified by biochemical experiments.

**Table 6:** The LigScore 2 values of the ten drugbank compounds that were selected for the MAO inhibition studies as well as their corresponding ROC coordinates

Rank	MAO-A	LigScore 2
5	Lamivudine	5.51
6	Emtricitabine	5.48
34	Zileuton	5.13
35	Proflavine	5.13
53	Thalidomide	5.05
74	Nitrofurantoin	4.98
92	Proguanil	4.93
96	Guanabenz	4.92 ^a^
	**MAO-B**	
1	Proguanil	4.89
36	Topiroxostat	5.10
96	Chlorpropamide	4.84 ^b^

^a^According to the ROC coordinates a LigScore 2 threshold value of 4.92 corresponds to a TPF of 0.143 and a FPF of 0.017

^b^According to the ROC coordinates a LigScore 2 threshold value of 4.84 corresponds to a TPF of 0.542 and a FPF of 0.061

### Monoamine oxidase inhibition studies

**Fig. 3:**
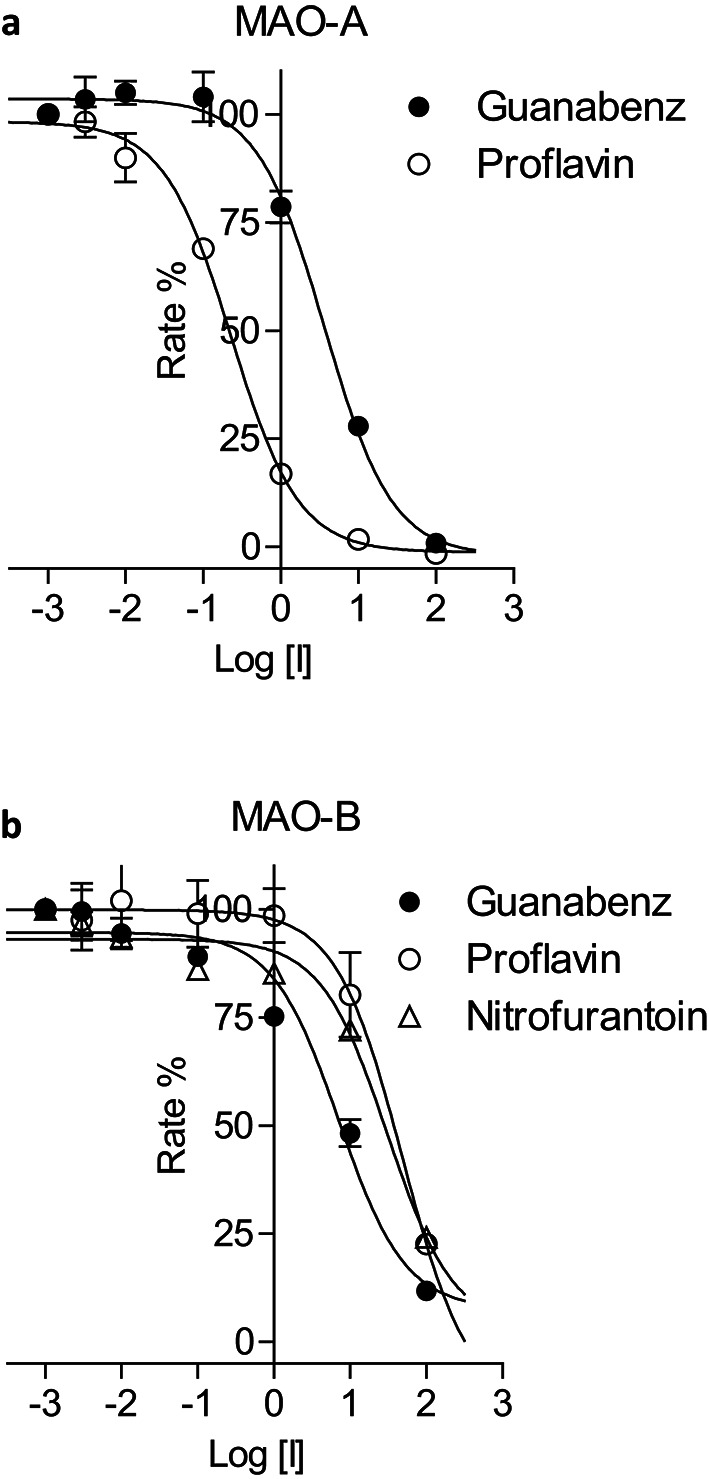
Sigmoidal graphs for the inhibition of MAO-A (panel **a**) and MAO-B (panel **b**) by proflavine, guanabenz and nitrofurantoin

The ten compounds that were selected as potential inhibitors of MAO-A and MAO-B were evaluated as in vitro inhibitors of the human MAO isoforms [32, 42]. The literature procedure was followed which uses kynuramine as substrate for both MAO-A and MAO-B. Kynuramine is oxidised by the MAO enzymes to yield a fluorescent metabolite, 4-hydroxyquinoline. Enzyme reactions containing the MAO enzymes, substrate and various concentrations of the test inhibitors (0.003–100 µM) were prepared and incubated for 20 min whereafter the reactions were terminated with the addition of sodium hydroxide. The concentration of 4-hydroxyquinioline that was produced by the action of the MAOs on kynuramine was measured by fluorescence spectrophotometry and the enzyme catalytic rates were calculated. The rate data were used to construct sigmoidal graphs of catalytic rate versus inhibitor concentration (log[I]) from which the IC_50_ values were determined (Fig. 3). These IC_50_ values were determined in triplicate and are presented in Table 7. Among the ten compounds evaluated, only proflavine and guanabenz presented with good potency (IC_50_ < 10 µM) MAO-A inhibition with IC_50_ values of 0.223 and 3.46 µM, respectively. Proflavine may be viewed as a potent MAO-A inhibitor and was significantly more potent than the reference inhibitor, toloxatone (IC_50_ = 1.67 µM), while the inhibition potency of guanabenz was in the same range as toloxatone. Both proflavine and guanabenz were predicted by the virtual screening study to be MAO-A inhibitors. The only good potency MAO-B inhibitor was guanabenz with an IC_50_ value of 8.49 µM. Although the virtual screening study did not place this compound within the 100 highest ranked compounds, it was ranked 435th with a LigScore 2 value of 4.24. According to the coordinates of the ROC curve this value corresponded to a TPF of 0.750 and a FPF of 0.137.

**Table 7:** The IC_50_ values (µM) for inhibition of human MAO-A and MAO-B by the ten drugbank compounds as well as reference inhibitors

	MAO-A (µM ± SD)^a^	MAO-B (µM ± SD)^a^	SI^b^
Lamivudine	NI^c^	NI^c^	–
Emtricitabine	NI^c^	NI^c^	–
Zileuton	NI^c^	NI^c^	–
Proflavine	0.223 ± 0.005	34.3 ± 5.85	154
Thalidomide	NI^c^	NI^c^	–
Nitrofurantoin	62.8 ± 0.650	32.9 ± 0.73	0.5
Proguanil	NI^c^	NI^c^	
Guanabenz	3.46 ± 0.216	8.49 ± 1.02	2.5
Topiroxostat	59.9 ± 2.81	NI^c^	–
Chlorpropamide	NI^c^	NI^c^	–
Toloxatone^d^	1.67 ± 0.418	–	–
Safinamide^d^	*–*	0.240 ± 0.080	–

^a^Reported as the mean ± standard deviation (SD) of triplicate determinations

^b^SI, selectivity index is calculated as IC_50_(MAO-B)/IC_50_(MAO-A)

^c^No inhibition observed at a maximal tested concentration of 100 µM

^d^Reference inhibitors

**Fig. 4:**
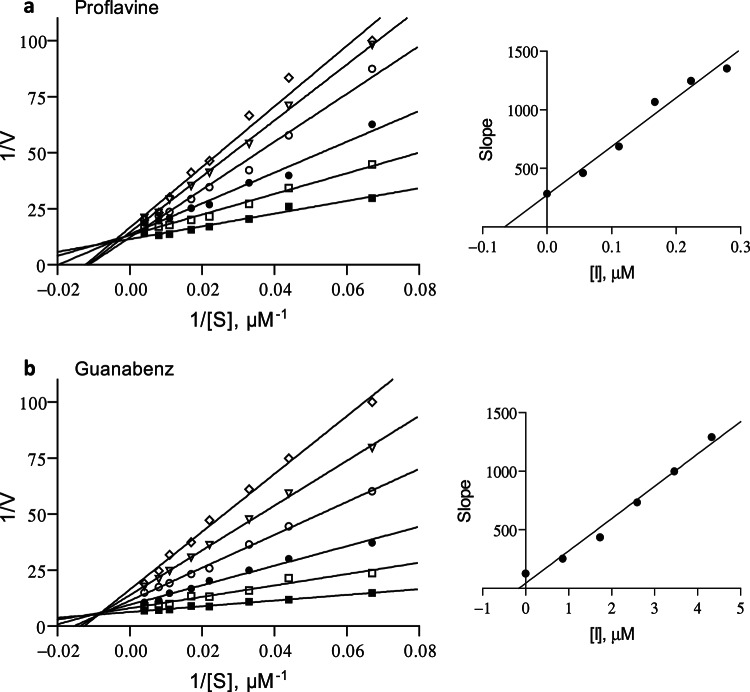
Lineweaver-Burk graphs for the inhibition of MAO-A by proflavine (panel **a**) and guanabenz (panel **b**). The insets are replots of the slopes of the Lineweaver-Burk graphs versus inhibitor concentration from which a K_i_ value of 0.066 and 0.16 µM were estimated for proflavine and guanabenz, respectively

Based on the good MAO-A inhibition potencies of proflavine and guanabenz, their mechanisms of inhibition were further investigated by constructing Lineweaver-Burk graphs. For these experiments the concentration of kynuramine ranged from 15 to 250 µM while the concentrations of the inhibitors were 0.0558–0.279 µM for proflavin and 0.865–4.325 µM for guanabenz. For both proflavine and guanabenz, the lines of the Lineweaver-Burk graphs were linear and intercepted on a single point near the y-axis (Fig. 4). This behaviour was indicative of competitive inhibition. Replots of the slopes of the Lineweaver-Burk graphs versus inhibitor concentration yielded linear lines from which the K_i_ values were estimated. These were 0.066 and 0.16 µM for proflavine and guanabenz, respectively.

### Binding orientations and interactions of proflavine and guanabenz

**Fig. 5:**
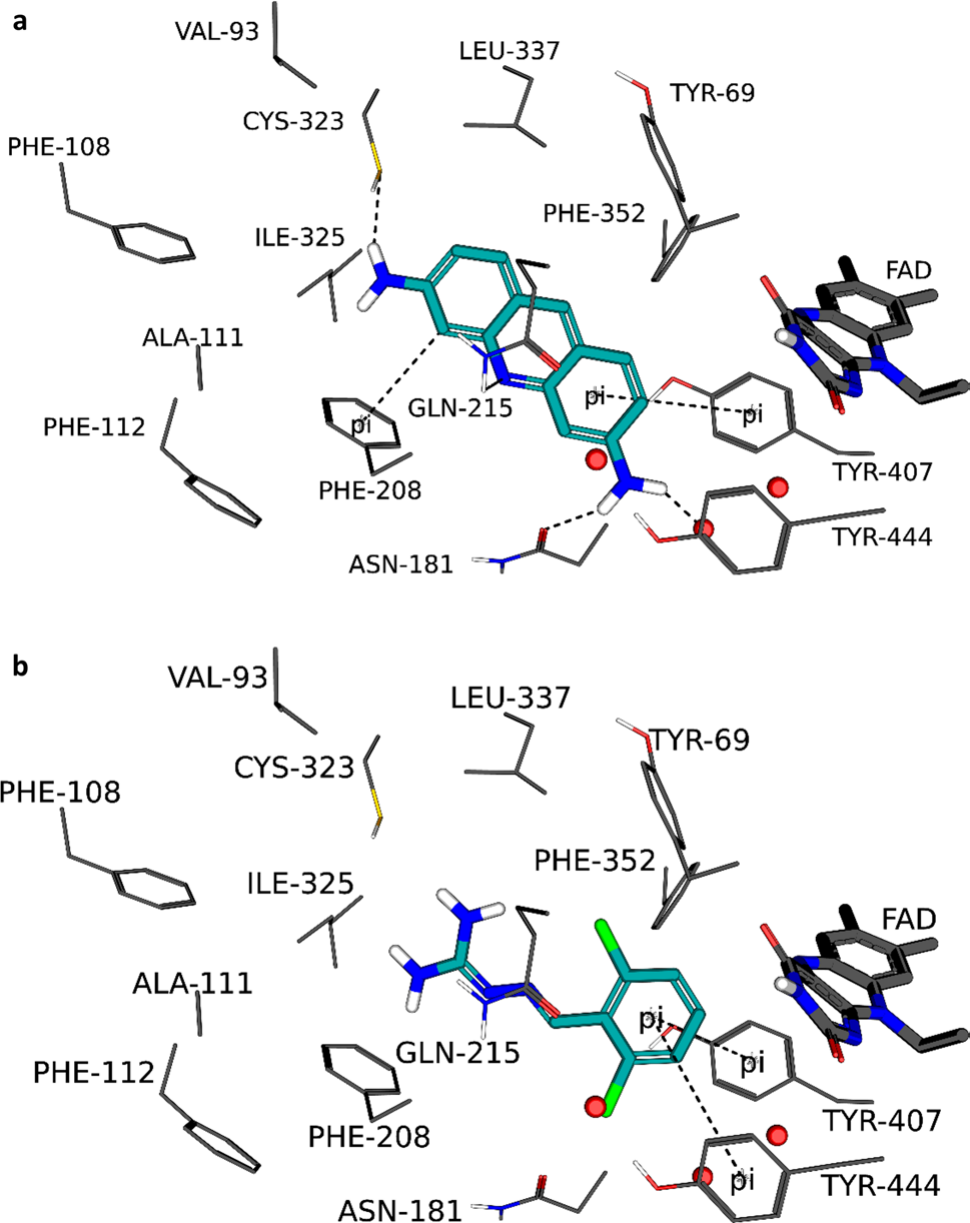
The binding orientations of the highest ranked poses of proflavine (panel **a**) and guanabenz (panel **b**) to the active site of MAO-A as predicted by LibDock/LigScore 2. The FAD cofactors are shown as sticks (dark grey), the residues lining the active sites are shown as lines and the water molecules are indicated by red spheres. The ligands are indicated as cyan sticks and hydrogen bonding and other interactions by dashed lines

**Fig. 6:**
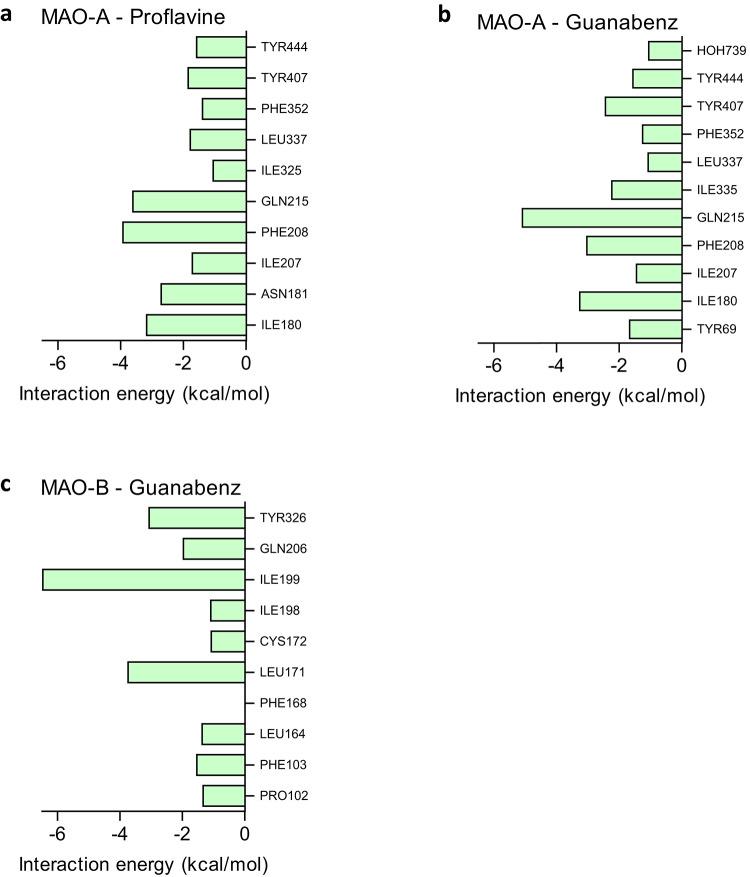
Estimates of the interaction energies of proflavine and guanabenz with the active site residues of MAO-A and MAO-B. Only energies more negative than − 1 kcal/mol are shown

The MAO inhibition studies found that proflavine and guanabenz were good potency MAO-A inhibitors, while guanabenz was identified as an MAO-B inhibitor. The highest ranked poses for proflavine and guanabenz bound to MAO-A, as predicted by the LibDock/LigScore 2 combination, are presented in Fig. 5. Proflavine was predicted to bind in proximity to the FAD co-factor with the primary amine group placed in the space in front of the aromatic sandwich residues, Tyr407 and TYR444. The inhibitor formed hydrogen bond interactions with the side chains of Asn181 and Gln215 as well as with a water molecule. A pi-pi interaction occurred between the Tyr407 and the proximal phenyl of the inhibitor, while a pi-sigma interaction occurred between the distal phenyl ring and Phe208. There also was a productive polar interaction between the side chain of Cys323 and the nearby amine of the inhibitor. For the interaction of proflavine with MAO-A, Ile180, Asn181, Phe208 and Gln215 contributed most to the stabilization of the complex (Fig. 6). The total interaction energy for the complex between MAO-A and proflavine was − 28.9 kcal/mol.

Guanabenz, in turn, was placed with the dichlorophenyl moiety in proximity to the FAD co-factor while the guanidine group extended towards the entrance of the MAO-A active site. While no hydrogen bond interactions were predicted for the complex, the phenyl formed pi-pi interactions with Tyr407 and Tyr444. For the interaction of guanabenz with MAO-A, Ile180, Phe208, Gln215 and Tyr407 contributed most to the stabilization of the complex with a total interaction energy of − 28.0 kcal/mol.

**Fig. 7:**
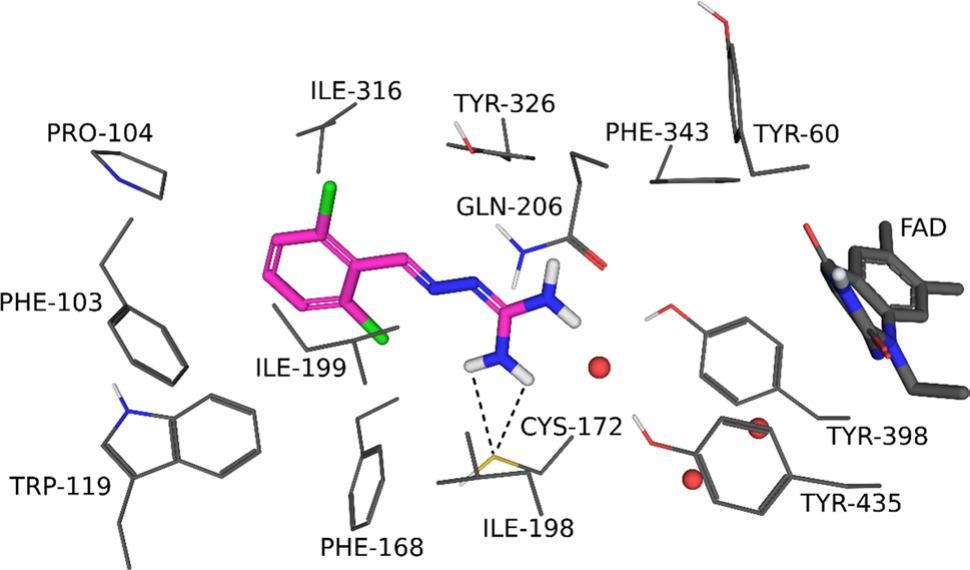
The binding orientation of the highest ranked pose of guanabenz to the active site of MAO-B as predicted by LibDock/LigScore 2. The FAD cofactor is shown as sticks (dark grey), the residues lining the active site are shown as lines and the water molecules are indicated by red spheres. The ligand is indicated as magenta sticks and interactions by dashed lines

The binding orientation of guanabenz to MAO-B as predicted by the LibDock/LigScore 2 combination is presented in Fig. 7. In contrast to MAO-A, the dichlorophenyl moiety was placed in the entrance cavity of MAO-B, directed away from the FAD co-factor. The guanidine group was directed towards the FAD co-factor, but placed at a distance from the FAD, leaving the substrate cavity partly unoccupied. As a result, no hydrogen bond interactions were observed for the complex, while a productive polar interaction between the side chain of Cys172 and the nearby amine of the inhibitor was observed. An analysis of the interactions between guanabenz and MAO-B showed that the following residues contributed most to the interaction energy with the enzyme: Leu171, Ile199, Gln206 and Tyr326. The total interaction energy for the complex between MAO-B and guanabenz was − 17.9 kcal/mol, which was more positive than the value recorded for the complex with MAO-A. This was in agreement with the lower inhibition potency for MAO-B compared to MAO-A observed for this compound.

## Conclusion

**Fig. 8:**
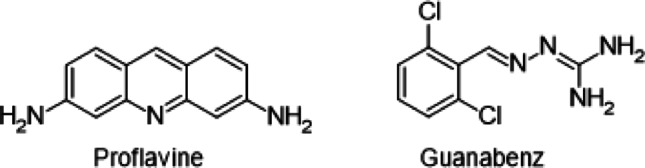
The structures of proflavine and guanabenz

This study employed a molecular docking approach to discover new MAO inhibitors. To improve to probability of success, the virtual screening protocol was validated by finding the combinations of docking and scoring functions that most accurately identified known MAO inhibitors. For the validation, the EF^10%^ and ROC-AUC were evaluated, and the LibDock/LigScore 2 combination was found to give accurate results for both MAO-A and MAO-B. This combination also performed well at predicting the correct orientations of co-crystallized ligands when redocked into the active sites of the MAO enzymes as judged by the RMSD values. The compounds listed in the DrugBank were subsequently docked and scored with the LibDock/LigScore 2 combination. Among the top 100 hits for MAO-A and MAO-B, ten compounds were purchased and evaluated as in vitro inhibitors of human MAO-A and MAO-B. Guanabenz (IC_50_ = 3.46 µM) and proflavine (IC_50_ = 0.223 µM) were found to be the most potent MAO-A inhibitors. Guanabenz also proved to be an MAO-B inhibitor with an IC_50_ value of 8.49 (Fig. 8).

Virtual screening of the compounds of the DrugBank to discover new active compounds for a specific target is often the first step in a drug repurposing program. Drug repurposing has the advantage that, in many instances, the safety and pharmacokinetic profiles of the drugs have been evaluated in humans. This gives the researcher valuable information regarding the likelihood that an active compound would be a viable drug in the clinical setting. Proflavine is a well-known antibacterial agent and effective disinfectant. Based on its mutagenic activity, proflavine and its derivatives has also been investigated as chemotherapeutic agents in cancer [43]. The use of proflavine is now limited due to toxicity and carcinogenicity concerns in mammals and proflavine is considered to be mainly an investigational drug [44]. Proflavine is a planar aromatic structure that can intercalate within double-stranded DNA. This process interferes with many key cellular functions and forms the basis of the biological activity of proflavine [43]. Hoffmann and colleagues reported the mutagenic activity of proflavine using a *Saccharomyces cerevisiae* strain. The study provided data that proflavine, a potent frameshift mutagen, can potentiate the mutagenic effects of other agents like bleomycin by increasing DNA accessibility [45]. An earlier study indicated that proflavine can intercalate between DNA basepairs to induce direct frameshift [46]. While proflavine may be systemically too toxic to be repurposed as an MAO inhibitor for neuropsychiatric and neurodegenerative disorders, it is noteworthy that MAO-A inhibition is being investigated as a potential treatment for prostate cancer [47–49]. Proflavine may have a dual mechanism is this regard, potent MAO-A inhibition as well as intercalation and stabilisation of DNA-topoisomerase II intermediates. Interestingly, close structural derivatives of proflavine have been reported to inhibit the MAO enzymes. For example, methylene blue is a potent MAO-A inhibitor with an IC_50_ value of 0.07 µM while 9-aminoacridine and 9-chloroacridine inhibit MAO-A with IC_50_ values of 26 and 12.1 µM, respectively [50, 51]. Similar to proflavine, these compounds are more specific for the MAO-A isoform.

The other MAO inhibitor identified in this study, guanabenz, is a selective agonist at α_2_-adrenergic receptors for the treatment of hypertension [52]. Interestingly, guanabenz has a hydrazine structure similar to the older MAO inhibitors such as phenelzine and isocarboxazid, and MAO inhibition for guanabenz has in fact been described before. Guanabenz was shown to be a moderate inhibitor of rat liver MAO-A with an IC_50_ value of 4 µM, which is in the same range as the value recorded in this study [53]. Furthermore, guanabenz demonstrated competitive inhibition of MAO-A, which is similar to the current study. MAO-A inhibition by guanabenz may counter its centrally mediated hypotensive activity since MAO-A inhibitors, particularly irreversibly acting inhibitors, might enhance systemic concentrations of tyramine and other sympathomimetic amines. This will have the effect of increased blood-pressure and has been termed the cheese effect [2]. Based on the analysis above, neither proflavine nor guanabenz would be good candidates for repurposing as MAO inhibitors for the treatment of neuropsychiatric and neurodegenerative disorders. Although virtual screening has limitations, the protocol validated here can be used to screen larger compound databases of diverse synthetic compounds. It is anticipated that new classes of MAO inhibitors might be discovered in this manner. In addition, the proposal that proflavine may possess a dual mechanism of action in the treatment of cancer merits further investigation. In this regard, structural analogues of proflavine should be explored as potential dual MAO-A inhibitors and DNA-intercalating drugs. In particular dye compounds such as methylene blue and others (e.g., cresyl violet, Nile blue, 1,9-dimethyl methylene blue, acridine orange, Darrow red) that have been shown to act as potent MAO-A inhibitors are planar structures and might thus interact with DNA basepairs [54, 55].

## Supplementary Information

Below is the link to the electronic supplementary material.

Supplementary Material 1

## Data Availability

Data is provided within the manuscript or supplementary information files.

## Abbreviations

DUD-E: Directory of useful decoys: enhanced
EF^10%^: Enrichment factor
FAD: Flavin adenine dinucleotide
FPF: False positive fractions
MAO: Monoamine oxidase
PDB: Protein data bank
RMSD: Root mean square deviation
ROC-AUC: Area under the receiver operating characteristic curve
SD: Standard deviation
SI: Selectivity index
TPF: True positive fractions
