# Supplementary material for: The discovery of monoamine oxidase inhibitors: virtual screening and in vitro inhibition potencies
Source: J Comput Aided Mol Des. 2026 Jan 31;40(1):55. doi: 10.1007/s10822-026-00764-y (PMC12860832; doi:10.1007/s10822-026-00764-y)
Supplement: Supplementary file 1 — Supplementary Material 1 [file 10822_2026_764_MOESM1_ESM.pdf]

Journal of Computer-Aided Molecular Design

The discovery of monoamine oxidase inhibitors: Virtual screening and *in vitro* inhibition potencies

Maryké Shaw[1] · Anél Petzer[1,2] · Chantalle Crous[1] · Theunis T. Cloete[1,2] · Jacobus P. Petzer[1,2]

[1] Centre of Excellence for Pharmaceutical Sciences, North-West University, Private Bag X6001, Potchefstroom 2520, South Africa

[2] Pharmaceutical Chemistry, School of Pharmacy, North-West University, Private Bag X6001, Potchefstroom 2520, South Africa

Corresponding author:

✉ Jacobus P. Petzer

jacques.petzer@nwu.ac.za

Tel.: +27 18 299 2262

Table S1 The 23 known inhibitors of MAO-A and 24 known MAO-B inhibitors that were selected from the literature to determine $EF^{10\%}$ and ROC-AUC values

**MAO-A Inhibitors**				
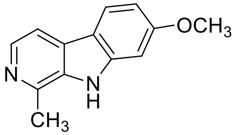<br>MAO-A: IC_{50} = 0.0041 µM<br>MAO-B: IC_{50} = NI (Myburg *et al.*, 2022)	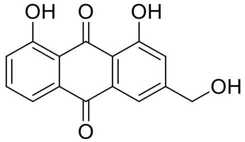<br>MAO-A: IC_{50} = 1.77 µM<br>MAO-B: IC_{50} = 21.9 µM (Lefin *et al.*, 2022)	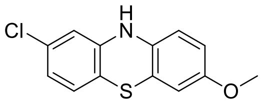<br>MAO-A: IC_{50} = 0.576 µM<br>MAO-B: IC_{50} = 1.34 µM (Lefin *et al.*, 2022)	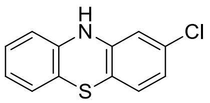<br>MAO-A: IC_{50} = 1.22 µM<br>MAO-B: IC_{50} = NI (Lefin *et al.*, 2022)	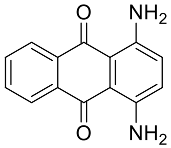<br>MAO-A: IC_{50} = 1.06 µM<br>MAO-B: IC_{50} = NI (Lefin *et al.*, 2022)
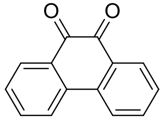<br>MAO-A: IC_{50} = 1.92 µM<br>MAO-B: IC_{50} = NI (Lefin *et al.*, 2022)	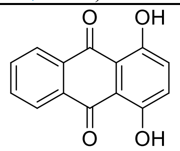<br>MAO-A: IC_{50} = 0.065 µM<br>MAO-B: IC_{50} = 110 µM (Lefin *et al.*, 2022)	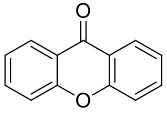<br>MAO-A: IC_{50} = 0.623 µM<br>MAO-B: IC_{50} = NI (Lefin *et al.*, 2022)	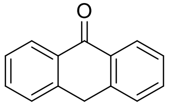<br>MAO-A: IC_{50} = 1.89 µM<br>MAO-B: IC_{50} = NI (Lefin *et al.*, 2022)	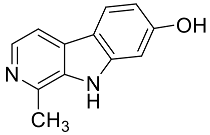<br>MAO-A: IC_{50} = 0.211 µM<br>MAO-B: IC_{50} = NI (Myburg *et al.*, 2022)
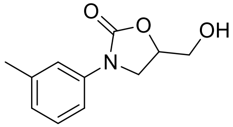<br>MAO-A: IC_{50} = 1.67 µM<br>MAO-B: IC_{50} = NI (Stear *et al.*, 2024)	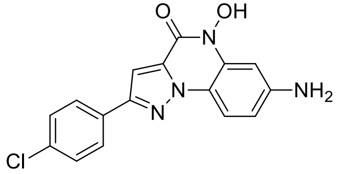<br>MAO-A: IC_{50} = 0.345 µM<br>MAO-B: IC_{50} = NI (Panova *et al.*, 2021)	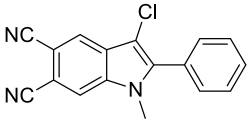<br>MAO-A: IC_{50} = 0.142 µM<br>MAO-B: IC_{50} = NI (Chirkova *et al.*, 2015)	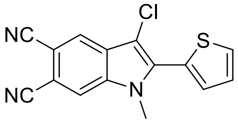<br>MAO-A: IC_{50} = 0.035 µM<br>MAO-B: IC_{50} = NI (Chirkova *et al.*, 2015)	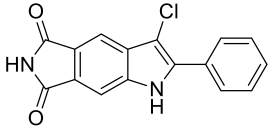<br>MAO-A: IC_{50} = 0.250 µM<br>MAO-B: IC_{50} = NI (Chirkova *et al.*, 2016)
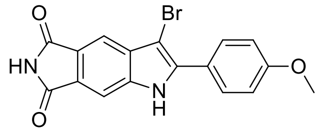<br>MAO-A: IC_{50} = 0.011 µM<br>MAO-B: IC_{50} = 6.64 µM (Chirkova *et al.*, 2019)	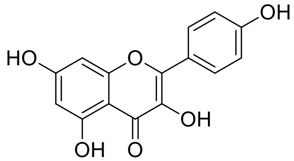<br>MAO-A: IC_{50} = 0.525 µM<br>MAO-B: IC_{50} = NI (Gidaro *et al.*, 2016)	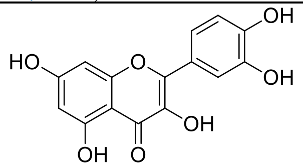<br>MAO-A: IC_{50} = 3.98 µM<br>MAO-B: IC_{50} = NI (Gidaro *et al.*, 2016)	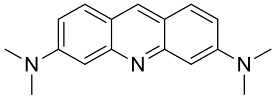<br>MAO-A: IC_{50} = 0.017 µM<br>MAO-B: IC_{50} = 12.4 µM (De Beer *et al.*, 2020)	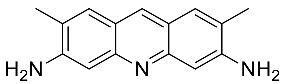<br>MAO-A: IC_{50} = 0.350 µM<br>MAO-B: IC_{50} = 11.9 µM (De Beer *et al.*, 2020)
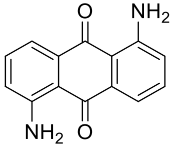<br>MAO-A: IC_{50} = 0.585 µM<br>MAO-B: IC_{50} = NI (De Beer *et al.*, 2020)	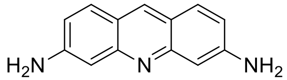<br>MAO-A: IC_{50} = 0.507 µM<br>MAO-B: IC_{50} = 32.5 µM (De Beer *et al.*, 2020)	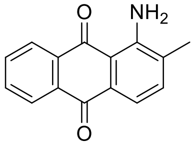<br>MAO-A: IC_{50} = 0.450 µM<br>MAO-B: IC_{50} = NI (De Beer *et al.*, 2020)		

**MAO-B Inhibitors**				
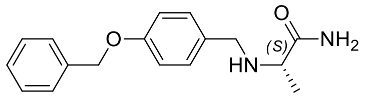<br>MAO-A: NI<br>MAO-B: IC_{50} = 0.240 µM (Stear *et al.*, 2024)	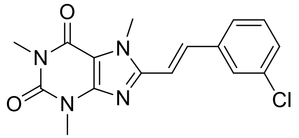<br>MAO-A: NI<br>MAO-B: IC_{50} = 0.070 µM (Chen *et al.*, 2002)	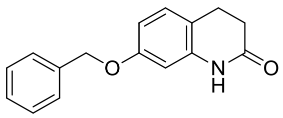<br>MAO-A: IC_{50} = 90.4 µM<br>MAO-B: IC_{50} = 0.038 µM (Meiring *et al.*, 2013)	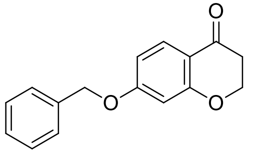<br>MAO-A: IC_{50} = NI<br>MAO-B: IC_{50} = 0.015 µM (Cloete *et al.*, 2021)	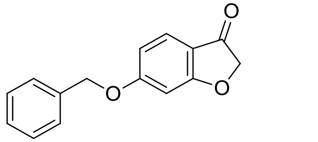<br>MAO-A: IC_{50} = 26.7 µM<br>MAO-B: IC_{50} = 0.062 µM (Van Dyk *et al.*, 2015)
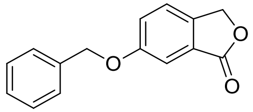<br>MAO-A: IC_{50} = 1.19 µM<br>MAO-B: IC_{50} = 0.024 µM (Strydom *et al.*, 2013)	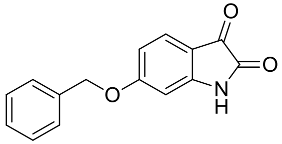<br>MAO-A: IC_{50} = 72.4 µM<br>MAO-B: IC_{50} = 0.138 µM (Manley-King *et al.*, 2011a)	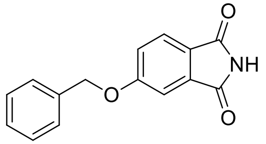<br>MAO-A: IC_{50} = 4.17 µM<br>MAO-B: IC_{50} = 0.043 µM (Manley-King *et al.*, 2011b)	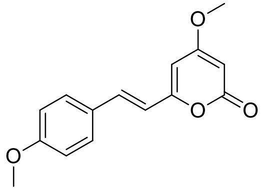<br>MAO-A: IC_{50} = 1.29 µM<br>MAO-B: IC_{50} = 0.085 µM (Prinsloo *et al.*, 2019)	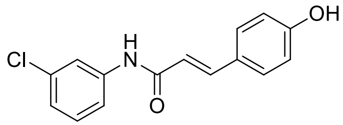<br>MAO-A: IC_{50} = 47.3 µM<br>MAO-B: IC_{50} = 0.032 µM (Legoabe *et al.*, 2011)
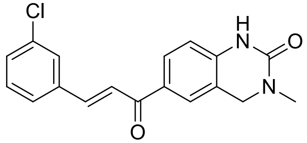<br>MAO-A: IC_{50} = 44.2 µM<br>MAO-B: IC_{50} = 0.350 µM (Marais *et al.*, 2020)	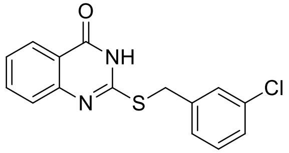<br>MAO-A: IC_{50} = NI<br>MAO-B: IC_{50} = 0.230 µM (Qhobosheane *et al.*, 2018)	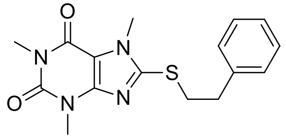<br>MAO-A: IC_{50} = 18.7 µM<br>MAO-B: IC_{50} = 0.271 µM (Mostert *et al.*, 2012)	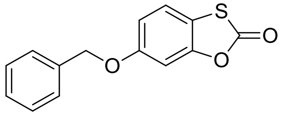<br>MAO-A: IC_{50} = 5.41 µM<br>MAO-B: IC_{50} = 0.051 µM (Mostert *et al.*, 2016)	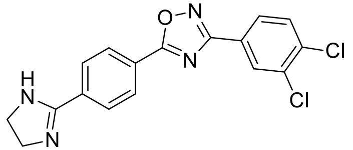<br>MAO-A: IC_{50} = 1.11 µM<br>MAO-B: IC_{50} = 0.012 µM (Shetnev *et al.*, 2019a)
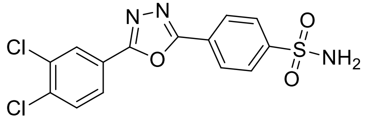<br>MAO-A: IC_{50} = 46.2 µM<br>MAO-B: IC_{50} = 0.0027 µM (Shetnev *et al.*, 2019b)	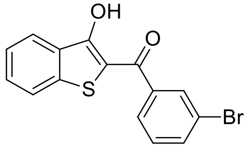<br>MAO-A: IC_{50} = 63.2 µM<br>MAO-B: IC_{50} = 0.35 µM (Guglielmi *et al.*, 2019)	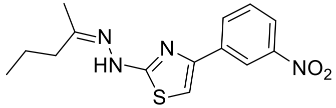<br>MAO-A: IC_{50} = 38.0 µM<br>MAO-B: IC_{50} = 0.055 µM (Carradori *et al.*, 2018)	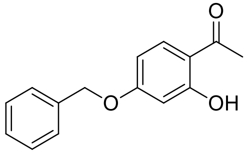<br>MAO-A: IC_{50} = 8.36 µM<br>MAO-B: IC_{50} = 0.007 µM (Legoabe *et al.*, 2015)	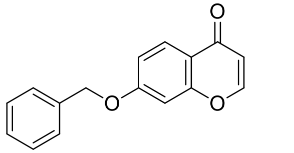<br>MAO-A: IC_{50} = 5.33 µM<br>MAO-B: IC_{50} = 0.085 µM (Legoabe *et al.*, 2012)
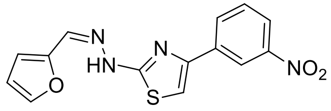<br>MAO-A: IC_{50} = 8.98 µM<br>MAO-B: IC_{50} = 0.095 µM (Secci *et al.*, 2019)	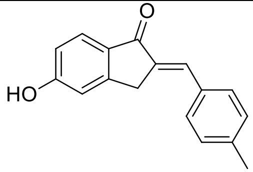<br>MAO-A: IC_{50} = 3.22 µM<br>MAO-B: IC_{50} = 0.0052 µM (Nel *et al.*, 2016)	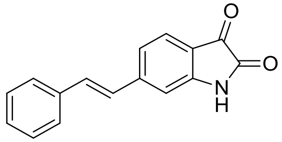<br>MAO-A: IC_{50} = 22 µM<br>MAO-B: IC_{50} = 0.56 µM (Van der Walt *et al.*, 2009)	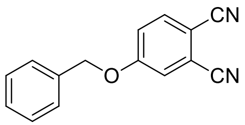<br>MAO-A: IC_{50} = 1.79 µM<br>MAO-B: IC_{50} = 0.0079 µM (Manley-King *et al.*, 2012)	

Table S2 RMSD as validation metric for redocking of co-crystallised ligands into the active site of MAO-B with *CDocker* and scoring the resulting poses with selected scoring functions

		**Scoring functions**										
**PDB Code**	**Best pose (rank)**	**Native - 1st** [c]	**LigScore 1 - 1st (rank)**	**LigScore 2 - 1st (rank)**	**PLP 1 - 1st (rank)**	**PLP 2 - 1st (rank)**	**Jain - 1st (rank)**	**PMF - 1st (rank)**	**PMF 4 - 1st (rank)**	**Ludi 1 - 1st (rank)**	**Ludi 2 - 1st (rank)**	**Ludi 3 - 1st (rank)**
**2V5Z**	0.4 (1)	0.4	0.4 (1)	0.4 (1)	0.4 (1)	0.4 (1)	0.4 (1)	1.6 (7)	1.6 (6)	0.4 (1)	0.4 (1)	0.4 (1)
**1OJ9**	0.7 (8)	1.0	0.8 (2)	0.8 (7)	1.0 (1)	1.0 (1)	0.8 (7)	0.8 (6)	1.0 (5)	0.8 (2)	0.8 (6)	0.8 (6)
**1OJA**	1.7 (8)	1.9	2.0 (10)	2.0 (10)	1.8 (5)	1.8 (5)	1.8 (6)	2.4 (9)	2.4 (9)	1.9 (1)	2.0 (10)	1.9 (1)
**1OJD**	2.2 (8)	8.7	2.6 (3)	2.6 (3)	2.6 (3)	9.2 (6)	2.2 (9)	2.6 (2)	2.6 (3)	2.6 (2)	2.3 (7)	2.2 (9)
**2BK3**	1.6 (8)	2.0	8.2 (10)	2.3 (3)	2.4 (7)	2.4 (7)	1.6 (8)	2.3 (3)	1.6 (8)	2.3 (3)	2.3 (3)	2.3 (3)
**2V60**	0.8 (7)	0.8	0.8 (9)	0.8 (9)	0.8 (2)	0.8 (10)	0.8 (1)	0.8 (9)	0.8 (10)	0.8 (10)	0.8 (2)	0.8 (1)
**2V61**	3.0 (4)	3.0	3.0 (3)	8.3 (9)	8.3 (9)	3.0 (3)	3.2 (8)	3.0 (7)	3.0 (3)	3.0 (6)	3.0 (5)	8.7 (10)
**3PO7**	1.5 (2)	2.7	2.7 (1)	5.2 (9)	1.5 (2)	1.5 (2)	8.0 (3)	5.6 (6)	1.5 (2)	2.7 (1)	2.7 (1)	2.7 (1)
**4A79**	3.0 (4)	3.0	3.5 (6)	3.4 (5)	3.0 (3)	3.0 (1)	3.5 (7)	3.0 (3)	3.0 (2)	3.5 (9)	3.4 (5)	3.3 (10)
**4A7A**	1.2 (6)	1.2	1.2 (1)	1.2 (6)	1.4 (4)	1.4 (4)	1.2 (2)	1.3 (8)	1.3 (5)	1.2 (7)	1.2 (3)	1.2 (2)
**4CRT**	3.2 (5)	8.0	8.0 (4)	8.2 (8)	3.2 (6)	3.2 (6)	3.4 (7)	3.2 (6)	3.2 (6)	3.2 (6)	8.0 (3)	8.0 (3)
**5MRL**	3.0 (3)	3.2	3.2 (1)	8.6 (7)	3.2 (1)	3.2 (1)	8.5 (5)	3.1 (6)	3.1 (6)	8.6 (7)	3.1 (6)	3.1 (6)
**6FVZ**	0.7 (7)	0.8	0.8 (6)	0.8 (6)	0.8 (6)	1.7 (10)	0.7 (7)	0.9 (4)	0.8 (6)	0.8 (3)	0.8 (6)	1.7 (10)
**6FW0**	2.1 (2)	2.2	2.2 (1)	2.2 (1)	7.9 (7)	7.9 (6)	2.2 (1)	2.1 (2)	7.9 (10)	2.1 (2)	7.9 (9)	7.9 (5)
**6FWC**	0.7 (6)	0.8	0.8 (9)	0.7 (4)	0.7 (6)	0.7 (5)	0.8 (7)	0.8 (1)	0.8 (2)	0.7 (4)	0.8 (7)	0.7 (4)
**6RKB**	2.2 (6)	2.4	2.4 (1)	2.4 (1)	2.4 (10)	2.4 (10)	2.4 (3)	2.2 (6)	2.2 (6)	2.6 (8)	2.2 (6)	2.4 (3)
**6RKP**	1.8 (10)	1.9	2.0 (9)	2.0 (9)	2.0 (9)	2.0 (7)	1.9 (3)	1.8 (5)	2.0 (9)	1.8 (10)	2.0 (7)	2.0 (7)
**6RLE**	1.7 (4)	2.4	2.1 (8)	1.7 (4)	2.4 (2)	2.2 (5)	2.2 (3)	2.1 (8)	2.1 (8)	2.1 (6)	2.2 (7)	2.1 (6)
**6YT2**	4.9 (9)	4.9	4.9 (2)	4.9 (10)	4.9 (1)	4.9 (1)	4.9 (2)	4.9 (10)	4.9 (9)	4.9 (1)	4.9 (2)	4.9 (2)
**7B0V**	0.5 (8)	8.5	8.5 (2)	8.5 (1)	8.4 (6)	8.5 (1)	8.5 (3)	0.6 (10)	8.4 (6)	8.4 (4)	8.4 (4)	0.5 (7)
**7B0Z**	0.7 (4)	0.7	0.7 (4)	0.7 (4)	0.8 (10)	0.7 (4)	0.8 (8)	0.8 (10)	0.7 (4)	0.7 (2)	0.8 (10)	0.7 (2)
**Total** [a]	**13**	**9**	**9**	**10**	**10**	**9**	**10**	**9**	**10**	**9**	**8**	**9**
**Ave. RMSD**	**1.8**	**2.9**	**2.9**	**3.2**	**2.9**	**3.0**	**2.9**	**2.2**	**2.6**	**2.6**	**2.9**	**2.8**
**Success rate** [b]	**62**	**43**	**43**	**48**	**48**	**43**	**48**	**43**	**48**	**43**	**38**	**43**

[a] Number of ligands with an RMSD value < 2 Å

[b] Percentage of ligands with and RMSD value < 2 Å

[c] Native scoring function for CDOCKER – CDOCKER Energy

Table S3 RMSD as validation metric for redocking of co-crystallised ligands into the active site of MAO-B with *LibDock* and scoring the resulting poses with selected scoring functions

		Scoring functions										
**PDB Code**	**Best pose (rank)**	**Native - 1st** [c]	**LigScore 1 - 1st (rank)**	**LigScore 2 - 1st (rank)**	**PLP 1 - 1st (rank)**	**PLP 2 - 1st (rank)**	**Jain - 1st (rank)**	**PMF - 1st (rank)**	**PMF 4 - 1st (rank)**	**Ludi 1 - 1st (rank)**	**Ludi 2 - 1st (rank)**	**Ludi 3 - 1st (rank)**
**2V5Z**	1.0 (60)	2.0	1.2 (12)	1.2 (49)	1.4 (6)	9.5 (20)	9.2 (82)	2.3 (33)	2.3 (33)	1.7 (19)	1.2 (12)	9.2 (40)
**1OJ9**	1.2 (64)	3.1	2.6 (21)	2.6 (21)	2.6 (11)	2.3 (10)	2.0 (87)	1.2 (64)	1.5 (85)	1.7 (60)	2.1 (84)	3.0 (2)
**1OJA**	1.2 (6)	2.0	2.0 (1)	2.0 (1)	2.0 (1)	2.5 (4)	10.0 (22)	3.9 (2)	3.9 (2)	3.2 (3)	3.2 (3)	3.2 (3)
**1OJD**	1.9 (10)	1.9	2.6 (36)	9.6 (12)	1.9 (1)	9.4 (6)	3.0 (37)	9.6 (20)	2.7 (78)	2.3 (30)	3.0 (37)	2.1 (3)
**2BK3**	1.5 (17)	3.0	2.2 (6)	8.5 (21)	2.2 (6)	8.9 (4)	2.5 (7)	2.2 (6)	1.5 (17)	2.4 (43)	2.4 (43)	2.4 (43)
**2V60**	1.0 (11)	1.9	1.0 (11)	1.0 (11)	2.2 (8)	2.2 (5)	2.0 (53)	1.0 (11)	2.2 (5)	1.4 (45)	3.1 (25)	3.0 (73)
**2V61**	1.0 (16)	2.2	3.2 (14)	8.2 (25)	8.2 (25)	8.2 (25)	3.4 (47)	1.0 (16)	3.5 (9)	3.5 (20)	3.2 (50)	3.9 (3)
**3PO7**	1.5 (26)	1.8	2.1 (21)	2.1 (21)	4.2 (3)	4.3 (7)	7.1 (69)	4.3 (34)	1.7 (17)	4.2 (3)	4.2 (3)	4.3 (34)
**4A79**	1.7 (3)	4.0	4.0 (1)	4.0 (1)	4.0 (2)	3.2 (6)	9.7 (10)	4.0 (2)	4.0 (1)	3.2 (6)	3.2 (6)	4.0 (1)
**4A7A**	1.4 (45)	7.8	2.0 (25)	2.0 (25)	7.8 (1)	1.8 (26)	2.0 (44)	8.5 (63)	1.6 (19)	2.0 (25)	2.0 (25)	3.1 (88)
**4CRT**	2.6 (19)	2.8	8.0 (22)	8.0 (22)	7.9 (4)	7.9 (4)	8.3 (20)	3.7 (57)	8.0 (8)	2.8 (2)	8.4 (41)	4.2 (31)
**5MRL**	2.0 (41)	2.4	3.0 (26)	2.7 (34)	2.4 (1)	2.4 (3)	3.0 (26)	3.0 (21)	3.0 (21)	5.0 (80)	2.8 (77)	5.2 (81)
**6FVZ**	1.0 (1)	1.0	2.8 (17)	1.7 (4)	8.1 (2)	8.1 (2)	2.6 (21)	1.9 (10)	2.0 (22)	1.7 (6)	2.4 (8)	2.4 (8)
**6FW0**	0.9 (54)	1.0	1.4 (9)	1.0 (1)	1.0 (1)	2.5 (15)	7.7 (16)	1.4 (9)	1.0 (1)	7.7 (27)	7.7 (43)	2.1 (5)
**6FWC**	0.8 (1)	0.8	2.0 (33)	0.8 (1)	0.8 (1)	7.4 (3)	3.4 (58)	1.9 (14)	0.8 (1)	3.4 (58)	3.4 (58)	2.4 (18)
**6RKB**	2.5 (31)	3.1	3.2 (12)	3.2 (12)	8.8 (8)	8.8 (9)	2.6 (41)	2.6 (26)	2.6 (32)	8.6 (49)	8.6 (83)	8.5 (67)
**6RKP**	1.7 (84)	2.2	9.5 (10)	9.5 (10)	9.5 (10)	9.5 (10)	9.4 (7)	2.5 (13)	9.6 (2)	2.0 (74)	2.0 (92)	9.6 (23)
**6RLE**	1.7 (98)	2.6	2.3 (3)	2.3 (3)	9.0 (6)	9.0 (6)	3.0 (76)	2.6 (9)	2.6 (9)	2.8 (27)	9.5 (14)	2.6 (1)
**6YT2**	1.6 (2)	5.0	4.8 (5)	4.7 (3)	1.6 (2)	1.6 (2)	7.4 (59)	2.3 (6)	5.2 (34)	5.0 (1)	7.4 (59)	2.3 (6)
**7B0V**	0.6 (23)	8.5	8.5 (7)	1.1 (34)	8.5 (2)	7.8 (20)	7.8 (20)	0.9 (57)	8.5 (7)	7.8 (20)	7.8 (20)	7.8 (20)
**7B0Z**	0.8 (23)	1.1	1.0 (15)	1.0 (24)	8.3 (38)	1.1 (11)	1.0 (18)	8.3 (34)	1.0 (18)	8.3 (41)	8.3 (31)	8.5 (49)
**Total** [a]	**19**	**8**	**6**	**8**	**5**	**3**	**1**	**7**	**8**	**6**	**2**	**0**
**Ave. RMSD**	**1.4**	**2.9**	**3.3**	**3.7**	**4.9**	**5.6**	**5.1**	**3.3**	**3.3**	**3.8**	**4.6**	**4.5**
**Success rate** [b]	**90**	**38**	**29**	**38**	**24**	**14**	**5**	**33**	**38**	**29**	**10**	**0**

[a] Number of ligands with an RMSD value < 2 Å
[b] Percentage of ligands with and RMSD value < 2 Å
[c] Native scoring function for LibDock – LibDock Score

Table S4 RMSD as validation metric for redocking of co-crystallised ligands into the active site of MAO-B with *LigandFit* and scoring the resulting poses with selected scoring functions

		**Scoring functions**										
**PDB Code**	**Best pose (rank)**	**Native - 1st** [c]	**LigScore 1 - 1st (rank)**	**LigScore 2 - 1st (rank)**	**PLP 1 - 1st (rank)**	**PLP 2 - 1st (rank)**	**Jain - 1st (rank)**	**PMF - 1st (rank)**	**PMF 4 - 1st (rank)**	**Ludi 1 - 1st (rank)**	**Ludi 2 - 1st (rank)**	**Ludi 3 - 1st (rank)**
**2V5Z**	1.3 (1)	1.3	1.3 (5)	1.3 (4)	9.2 (8)	9.2 (8)	1.3 (5)	1.3 (1)	1.3 (1)	1.3 (5)	1.3 (5)	9.2 (8)
**1OJ9**	0.6 (9)	0.7	0.9 (5)	0.9 (5)	0.9 (5)	0.9 (5)	0.9 (7)	0.7 (2)	0.8 (6)	0.7 (1)	0.9 (7)	0.7 (2)
**1OJA** [d]	–	–	–	–	–	–	–	–	–	–	–	–
**1OJD**	2.0 (1)	2.0	2.0 (1)	2.6 (10)	2.3 (7)	2.0 (2)	2.6 (8)	2.4 (5)	2.0 (1)	2.0 (2)	2.6 (8)	2.6 (8)
**2BK3**	8.3 (9)	8.7	8.7 (1)	8.7 (1)	8.7 (1)	8.7 (1)	8.5 (3)	8.6 (2)	8.6 (2)	8.4 (7)	8.4 (7)	8.4 (8)
**2V60**	0.9 (9)	2.2	2.2 (1)	2.2 (1)	2.2 (1)	2.2 (1)	2.1 (10)	1.0 (3)	0.9 (9)	1.9 (4)	2.2 (1)	2.2 (1)
**2V61**	3.1 (10)	8.1	3.2 (3)	8.1 (1)	3.1 (10)	3.3 (5)	8.4 (6)	3.1 (10)	3.1 (10)	3.3 (5)	3.1 (10)	8.1 (8)
**3PO7** [d]	–	–	–	–	–	–	–	–	–	–	–	–
**4A79**	3.3 (6)	3.6	3.6 (1)	3.6 (1)	3.4 (4)	3.4 (4)	3.6 (1)	3.6 (1)	3.6 (1)	3.5 (5)	3.4 (7)	3.6 (1)
**4A7A**	0.9 (9)	2.1	2.1 (2)	2.0 (3)	0.9 (9)	2.0 (4)	2.0 (4)	0.9 (6)	2.1 (1)	2.1 (1)	2.1 (2)	2.2 (7)
**4CRT**	7.9 (8)	8.0	8.0 (2)	8.0 (1)	8.0 (1)	8.0 (2)	8.0 (10)	8.0 (9)	7.9 (8)	8.0 (1)	8.1 (3)	8.1 (3)
**5MRL** [d]	–	–	–	–	–	–	–	–	–	–	–	–
**6FVZ**	0.7 (2)	0.7	0.7 (8)	0.7 (6)	0.7 (4)	0.7 (8)	2.0 (9)	0.7 (4)	0.7 (6)	2.0 (9)	2.0 (9)	0.7 (8)
**6FW0**	0.9 (10)	2.1	0.9 (9)	0.9 (9)	0.9 (9)	0.9 (9)	2.1 (4)	0.9 (9)	2.1 (2)	2.1 (1)	2.1 (1)	0.9 (9)
**6FWC**	7.5 (1)	7.5	7.5 (2)	7.5 (1)	7.5 (5)	7.5 (8)	7.5 (5)	7.5 (4)	7.5 (4)	7.5 (7)	7.5 (1)	7.5 (4)
**6RKB**	2.3 (1)	2.3	2.6 (10)	2.6 (10)	2.5 (6)	2.5 (6)	2.5 (4)	2.5 (2)	2.5 (2)	2.5 (2)	2.5 (8)	2.4 (3)
**6RKP**	1.8 (8)	2.1	9.4 (10)	9.4 (10)	2.3 (4)	2.3 (3)	9.4 (9)	1.8 (8)	9.4 (10)	2.1 (7)	2.1 (6)	2.3 (3)
**6RLE**	2.1 (3)	2.1	2.1 (1)	2.1 (1)	2.1 (1)	2.1 (2)	2.5 (6)	2.2 (4)	2.2 (4)	2.1 (1)	2.4 (10)	2.4 (10)
**6YT2** [d]	–	–	–	–	–	–	–	–	–	–	–	–
**7B0V**	0.7 (5)	0.8	0.9 (7)	0.9 (7)	0.7 (3)	0.7 (3)	1.0 (9)	0.8 (2)	0.8 (2)	0.8 (2)	0.9 (8)	0.8 (4)
**7B0Z**	8.0 (6)	8.1	8.1 (2)	8.1 (2)	8.0 (6)	8.0 (6)	8.1 (2)	8.1 (2)	8.1 (5)	8.1 (1)	8.1 (9)	8.1 (9)
**Total** [a]	**9**	**5**	**6**	**5**	**5**	**4**	**4**	**8**	**6**	**5**	**4**	**4**
**Ave. RMSD**	**2.5**	**3.0**	**3.8**	**4.1**	**3.7**	**3.8**	**4.3**	**3.2**	**3.8**	**3.4**	**3.5**	**4.1**
**Success rate** [b]	**43**	**24**	**29**	**24**	**24**	**19**	**19**	**38**	**29**	**24**	**19**	**19**

[a] Number of ligands with an RMSD value < 2 Å
[b] Percentage of ligands with and RMSD value < 2 Å
[c] Native scoring function for LigandFit – Dock Score
[d] No poses generated

Table S5 EF$^{10\%}$ and ROC-AUC values as validation metrics for identifying 24 known MAO-B inhibitors within the DUD-E and Schrödinger decoy datasets. Docking was carried out with *LigandFit* and the resulting poses were scored with selected scoring functions

	**LigandFit DUD-E** [b]		**LigandFit Schrödinger** [c]	
	**EF$^{10\%}$**	**ROC-AUC**	**EF$^{10\%}$**	**ROC-AUC**
**Native** [a]	8.72	0.89	8.72	0.839
**LigScore 1**	6.64	0.804	4.15	0.646
**LigScore 2**	8.72	0.897	8.72	0.887
**PLP 1**	8.30	0.892	1.66	0.684
**PLP 2**	6.64	0.843	1.66	0.57
**Jain**	0.83	0.346	0.00	0.239
**PMF**	8.30	0.847	0.42	0.511
**PMF 4**	5.40	0.777	2.08	0.563
**Ludi 1**	2.49	0.61	0.83	0.369
**Ludi 2**	2.49	0.536	0.42	0.382
**Ludi 3**	7.89	0.879	2.08	0.59

[a] Native scoring function for LigandFit – Dock Score

[b] Maximum EF$^{10\%}$ = 10.0 (2590 total compounds /24 active compounds)

[c] Maximum EF$^{10\%}$ = 10.0 (1046 total compounds /24 active compounds)

Table S6 $EF^{10\%}$ and ROC-AUC values as validation metrics for identifying 23 known MAO-A inhibitors within the DUD-E and Schrödinger decoy datasets. Docking was carried out with *LigandFit* and the resulting poses were scored with selected scoring functions

	**LigandFit DUD-E** [b]		**LigandFit Schrödinger** [c]	
	**$EF^{10\%}$**	**ROC-AUC**	**$EF^{10\%}$**	**ROC-AUC**
**Native** [a]	3.03	0.647	3.47	0.549
**LigScore 1**	3.46	0.708	3.47	0.57
**LigScore 2**	3.90	0.682	3.90	0.64
**PLP 1**	1.73	0.462	1.30	0.296
**PLP 2**	0.00	0.462	1.73	0.292
**Jain**	0.43	0.457	1.30	0.359
**PMF**	1.73	0.579	1.73	0.408
**PMF 4**	0.00	0.446	1.73	0.382
**Ludi 1**	1.73	0.559	2.17	0.459
**Ludi 2**	0.43	0.388	0.87	0.397
**Ludi 3**	3.03	0.804	2.60	0.567

[a] Native scoring function for LigandFit – Dock Score

[b] Maximum $EF^{10\%}$ = 10.0 (2590 total compounds /23 active compounds)

[c] Maximum $EF^{10\%}$ = 10.0 (1046 total compounds /23 active compounds)

References:

1. Carradori S, Ortuso F, Petzer A, Bagetta D, De Monte C, Secci D, De Vita D, Guglielmi P, Zengin G, Aktumsek A, Alcaro S, Petzer JP (2018) Design, synthesis and biochemical evaluation of novel multi-target inhibitors as potential anti-Parkinson agents. Eur J Med Chem 143:1543-1552. https://doi.org/10.1016/j.ejmech.2017.10.050

2. Chen JF, Steyn S, Staal R, Petzer JP, Xu K, Van Der Schyf CJ, Castagnoli K, Sonsalla PK, Castagnoli N, Jr., Schwarzschild MA (2002) 8-(3-Chlorostyryl)caffeine may attenuate MPTP neurotoxicity through dual actions of monoamine oxidase inhibition and A2A receptor antagonism. J Biol Chem 277(39):36040-36044. https://doi.org/10.1074/jbc.M206830200

3. Chirkova ZV, Kabanova MV, Filimonov SI, Abramov IG, Petzer A, Hitge R, Petzer JP, Suponitsky KY (2019) Optimization of pyrrolo[3,4]indole-5,7-dione and indole-5,6-dicarbonitrile derivatives as inhibitors of monoamine oxidase. Drug Develop Res 80(7):970-980. https://doi.org/10.1002/ddr.21576

4. Chirkova ZV, Kabanova MV, Filimonov SI, Abramov IG, Petzer A, Petzer JP, Firgang SI, Suponitsky KY (2015) Inhibition of monoamine oxidase by indole-5,6-dicarbonitrile derivatives. Bioorg Med Chem Lett 25(6):1206-1211. https://doi.org/10.1016/j.bmcl.2015.01.061

5. Chirkova ZV, Kabanova MV, Filimonov SI, Abramov IG, Petzer A, Petzer JP, Suponitsky KY (2016) An evaluation of synthetic indole derivatives as inhibitors of monoamine oxidase. Bioorg Med Chem Lett 26(9):2214-2219. https://doi.org/10.1016/j.bmcl.2016.03.060

6. Cloete SJ, N'Da CI, Legoabe LJ, Petzer A, Petzer JP (2021) The evaluation of 1-tetralone and 4-chromanone derivatives as inhibitors of monoamine oxidase. Mol Divers 25(1):491-507. https://doi.org/10.1007/s11030-020-10143-w

7. De Beer F, Petzer JP, Petzer A (2020) Monoamine oxidase inhibition by selected dye compounds. Chem Biol Drug Des 95(3):355-367. https://doi.org/10.1111/cbdd.13654

8. Gidaro MC, Astorino C, Petzer A, Carradori S, Alcaro F, Costa G, Artese A, Rafele G, Russo FM, Petzer JP, Alcaro S (2016) Kaempferol as selective human MAO-A inhibitor: Analytical detection in calabrian red wines, biological and molecular modeling studies. J Agr Food Chem 64(6):1394-1400. https://doi.org/10.1021/acs.jafc.5b06043

9. Guglielmi P, Secci D, Petzer A, Bagetta D, Chimenti P, Rotondi G, Ferrante C, Recinella L, Leone S, Alcaro S, Zengin G, Petzer JP, Ortuso F, Carradori S (2019) Benzo[b]tiophen-3-ol derivatives as effective inhibitors of human monoamine oxidase: design, synthesis, and biological activity. J Enzyme Inhib Med Chem 34(1):1511-1525. https://doi.org/10.1080/14756366.2019.1653864

10. Lefin R, Petzer A, Petzer JP (2022) Phenothiazine, anthraquinone and related tricyclic derivatives as inhibitors of monoamine oxidase. Bioorg Med Chem 54:116558. https://doi.org/10.1016/j.bmc.2021.116558

11. Legoabe L, Kruger J, Petzer A, Bergh JJ, Petzer JP (2011) Monoamine oxidase inhibition by selected anilide derivatives. Eur J Med Chem 46(10):5162-5174. https://doi.org/10.1016/j.ejmech.2011.08.036

12. Legoabe LJ, Petzer A, Petzer JP (2012) Selected C7-substituted chromone derivatives as monoamine oxidase inhibitors. Bioorg Chem 45:1-11. https://doi.org/10.1016/j.bioorg.2012.08.003

13. Legoabe LJ, Petzer A, Petzer JP (2015) 2-Acetylphenol analogs as potent reversible monoamine oxidase inhibitors. Drug Des Devel Ther 9:3635-3644. https://doi.org/10.2147/DDDT.S86225

14. Manley-King CI, Bergh JJ, Petzer JP (2011) Inhibition of monoamine oxidase by selected C5- and C6-substituted isatin analogues. Bioorg Med Chem 19(1):261-274. https://doi.org/10.1016/j.bmc.2010.11.028

15. Manley-King CI, Bergh JJ, Petzer JP (2011) Inhibition of monoamine oxidase by C5-substituted phthalimide analogues. Bioorg Med Chem 19(16):4829-4840. https://doi.org/10.1016/j.bmc.2011.06.070

16. Manley-King CI, Bergh JJ, Petzer JP (2012) Monoamine oxidase inhibition by C4-substituted phthalonitriles. Bioorg Chem 40(1):114-124. https://doi.org/10.1016/j.bioorg.2011.10.003

17. Marais L, Petzer A, Petzer JP, Legoabe LJ (2020) The monoamine oxidase inhibition properties of C6- and N1-substituted 3-methyl-3,4-dihydroquinazolin-2(1H)-one derivatives. Mol Divers 24(2):391-406. https://doi.org/10.1007/s11030-019-09960-5

18. Meiring L, Petzer JP, Petzer A (2013) Inhibition of monoamine oxidase by 3,4-dihydro-2(1H)-quinolinone derivatives. Bioorg Med Chem Lett 23(20):5498-5502. https://doi.org/10.1016/j.bmcl.2013.08.071

19. Mostert S, Mentz W, Petzer A, Bergh JJ, Petzer JP (2012) Inhibition of monoamine oxidase by 8-[(phenylethyl)sulfanyl]caffeine analogues. Bioorg Med Chem 20(24):7040-7050. https://doi.org/10.1016/j.bmc.2012.10.005

20. Mostert S, Petzer A, Petzer JP (2016) Inhibition of monoamine oxidase by benzoxathiolone analogues. Bioorg Med Chem Lett 26(4):1200-1204. https://doi.org/10.1016/j.bmcl.2016.01.034

21. Myburg T, Petzer A, Petzer JP (2022) The inhibition of monoamine oxidase by harmine derivatives. Results Chem 4:100607. https://doi.org/10.1016/j.rechem.2022.100607

22. Nel MS, Petzer A, Petzer JP, Legoabe LJ (2016) 2-Benzylidene-1-indanone derivatives as inhibitors of monoamine oxidase. Bioorg Med Chem Lett 26(19):4599-4605. https://doi.org/10.1016/j.bmcl.2016.08.067

23. Panova VA, Filimonov SI, Chirkova ZV, Kabanova MV, Shetnev AA, Korsakov MK, Petzer A, Petzer JP, Suponitsky KY (2021) Investigation of pyrazolo[1,5]quinoxalin-4-ones as novel monoamine oxidase inhibitors. Bioorg Chem 108: 104563. https://doi.org/10.1016/j.bioorg.2020.104563

24. Prinsloo D, van Dyk S, Petzer A, Petzer JP (2019) Monoamine oxidase inhibition by kavalactones from kava (Piper methysticum). Planta Med 85(14-15):1136-1142. https://doi.org/10.1055/a-1008-9491

25. Qhobosheane MA, Petzer A, Petzer JP, Legoabe LJ (2018) Synthesis and evaluation of 2-substituted 4(3H)-quinazolinone thioether derivatives as monoamine oxidase inhibitors. Bioorg Med Chem 26(20):5531-5537. https://doi.org/10.1016/j.bmc.2018.09.032

26. Secci D, Carradori S, Petzer A, Guglielmi P, D'Ascenzio M, Chimenti P, Bagetta D, Alcaro S, Zengin G, Petzer JP, Ortuso F (2019) 4-(3-Nitrophenyl)thiazol-2-ylhydrazone derivatives as antioxidants and selective hMAO-B inhibitors: Synthesis, biological activity and computational analysis. J Enzym Inhib Med Chem 34(1):597-612. https://doi.org/10.1080/14756366.2019.1571272

27. Shetnev A, Osipyan A, Baykov S, Sapegin A, Chirkova Z, Korsakov M, Petzer A, Engelbrecht I, Petzer JP (2019) Novel monoamine oxidase inhibitors based on the privileged 2-imidazoline molecular framework. Bioorg Med Chem Lett 29(1):40-46. https://doi.org/10.1016/j.bmcl.2018.11.018

28. Shetnev A, Shlenev R, Efimova J, Ivanovskii S, Tarasov A, Petzer A, Petzer JP (2019) 1,3,4-Oxadiazol-2-ylbenzenesulfonamides as privileged structures for the inhibition of monoamine oxidase B. Bioorg Med Chem Lett 29(21):126677. https://doi.org/10.1016/j.bmcl.2019.126677

29. Stear C, Petzer A, Crous C, Petzer JP (2024) Indazole derivatives as novel inhibitors of monoamine oxidase and D-amino acid oxidase. Med Chem Res 33(1):164-176. https://doi.org/10.1007/s00044-023-03176-x

30. Strydom B, Bergh JJ, Petzer JP (2013) Inhibition of monoamine oxidase by phthalide analogues. Bioorg Med Chem Lett 23(5):1269-1273. https://doi.org/10.1016/j.bmcl.2013.01.003

31. Van der Walt EM, Milczek EM, Malan SF, Edmondson DE, Castagnoli N, Jr., Bergh JJ, Petzer JP (2009) Inhibition of monoamine oxidase by (E)-styrylisatin analogues. Bioorg Med Chem Lett 19(9):2509-2513. https://doi.org/10.1016/j.bmcl.2009.03.030

32. Van Dyk AS, Petzer JP, Petzer A, Legoabe LJ (2015) 3-Coumaranone derivatives as inhibitors of monoamine oxidase. Drug Des Devel Ther 9:5479-5489. https://doi.org/10.2147/DDDT.S89961
